# GTSE1 is involved in breast cancer progression in p53 mutation-dependent manner

**DOI:** 10.1186/s13046-019-1157-4

**Published:** 2019-04-08

**Authors:** Fen Lin, Yu-Jie Xie, Xin-Ke Zhang, Tie-Jun Huang, Hong-Fa Xu, Yan Mei, Hu Liang, Hao Hu, Si-Ting Lin, Fei-Fei Luo, Yan-Hong Lang, Li-Xia Peng, Chao-Nan Qian, Bi-Jun Huang

**Affiliations:** 1 State Key Laboratory of Oncology in South China and Collaborative Innovation Center for Cancer Medicine, Sun Yat-sen University Cancer Center, Guangzhou, 510060 People’s Republic of China; 2 Guangdong Medical University, Zhanjiang, 524023 People’s Republic of China; 3 Department of pathology, Sun Yat-sen University Cancer Center, Guangzhou, 510060 People’s Republic of China; 4 Department of Nuclear Medicine, The Second People’s Hospital of Shenzhen, Shenzhen, People’s Republic of China; 5 Zhuhai Precision Medicine Center, Zhuhai People’s Hospital Affiliated with Jinan University, Zhuhai, Guangdong 519000 People’s Republic of China; 6 Department of Traditional Chinese Medicine, The First Affiliated Hospital of Sun Yat-Sen University, Guangzhou, 510060 People’s Republic of China; 7 Department of Nasopharyngeal Carcinoma, Sun Yat-Sen University Cancer Center, Guangzhou, 510060 People’s Republic of China

**Keywords:** Breast cancer, GTSE1, p53, Cell cycle, Prognosis

## Abstract

**Background:**

With the rapid development of the high throughput detection techniques, tumor-related Omics data has become an important source for studying the mechanism of tumor progression including breast cancer, one of the major malignancies worldwide. A previous study has shown that the G2 and S phase-expressed-1 (GTSE1) can act as an oncogene in several human cancers. However, its functional roles in breast cancer remain elusive.

**Method:**

In this study, we analyzed breast cancer data downloaded from The Cancer Genome Atlas (TCGA) databases and other online database including the Oncomine, bc-GenExMiner and PROGgeneV2 database to identify the molecules contributing to the progression of breast cancer. The GTSE1 expression levels were investigated using qRT-PCR, immunoblotting and IHC. The biological function of GTSE1 in the growth, migration and invasion of breast cancer was examined in MDA-MB-231, MDA-MB-468 and MCF7 cell lines. The in vitro cell proliferative, migratory and invasive abilities were evaluated by MTS, colony formation and transwell assay, respectively. The role of GTSE1 in the growth and metastasis of breast cancer were revealed by in vivo investigation using BALB/c nude mice.

**Results:**

We showed that the expression level of GTSE1 was upregulated in breast cancer specimens and cell lines, especially in triple negative breast cancer (TNBC) and p53 mutated breast cancer cell lines. Importantly, high GTSE1 expression was positively correlated with histological grade and poor survival. We demonstrated that GTSE1 could promote breast cancer cell growth by activating the AKT pathway and enhance metastasis by regulating the Epithelial-Mesenchymal transition (EMT) pathway. Furthermore, it could cause multidrug resistance in breast cancer cells. Interestingly, we found that GTSE1 could regulate the p53 function to alter the cell cycle distribution dependent on the mutation state of p53.

**Conclusion:**

Our results reveal that GTSE1 played a key role in the progression of breast cancer, indicating that GTSE1 could serve as a novel biomarker to aid in the assessment of the prognosis of breast cancer.

**Electronic supplementary material:**

The online version of this article (10.1186/s13046-019-1157-4) contains supplementary material, which is available to authorized users.

## Background

Breast cancer is one of the major malignancies worldwide [1, 2]. There are standardized therapies accessible for the Her2 and luminal subtypes, but for TNBC there are no standard treatment methods owing to its heterogeneity. [3–5]. Progress has been made in the treatment of breast cancer in recent decades, however its recurrence and metastasis remain the most common reasons for treatment failure [6]. In order to eliminate the bottleneck of breast cancer treatment, intensive research has been carried out in this field, but the molecular driving factors of tumor progression are still unclear [7]. Therefore, identifying the key molecular events associated with the malignant transformation of breast cancer cells and tumor progression is of great significance as it would allow the development of new solutions for the diagnosis, treatment and improvement of the patients’ survival.

The rapid development of high-throughput detection techniques has enabled the accumulation of tumor-related Omics data which are of great significance to study the mechanism of tumor progression [8, 9]. The management and mining of large quantities of biological data have become important in cancer research [10]. The TCGA database provides a large amount of tumor public data, which is widely used in tumor research, providing useful information for the discovery of new tumor biological indicators and drug targets [11, 12]. By taking advantages of bioinformatics, molecules related to the histological classification, recurrence and metastasis of breast cancer were screened to find out potential therapeutic targets which could benefit to the survival of breast cancer patients [13]. By analyzing public database, GTSE1 attracted our attention.

GTSE1 is located in chromosome 22q13.2-q13.3, which is expressed specifically during the G2 and S phases of the cell cycle [14]. A previous study has identified that the protein GTSE1 is mainly located in the cytoplasm and is associated with the activity of cytoplasmic tubulin and microtubules during mitosis [15]. It is a key regulator for chromosomal movement and spindle integrity during mitosis [16]. In addition, GTSE1 can act as a negative regulatory factor of p53 by accumulating in the nucleus and binding to p53 protein to transport it out of the nucleus for further degradation [17, 18]. Furthermore, overexpression of GTSE1 can lead to a delay in the transition of the G2 to M phase [19]. Further studies have shown that the expression level of GTSE1 was upregulated in different human cancers, while its high expression was not associated with lung cancer prognosis [20]. After treatment with cisplatin, GTSE1 was upregulated in myeloma cells, which showed a mechanism for clinical-acquired drug resistance [21]. GTSE1 was found to be overexpressed in HCC and could serve as a marker for poor prognosis as apart from reducing the sensitivity of HCC cells to 5-FU, it could also promote the malignant biological behavior of HCC by improving its proliferative and metastatic ability and upregulating the expression of EMT makers [22, 23]. In addition, it conferred to cisplatin resistance by the inhibition of p53 apoptotic signaling in gastric cancer cells [24]. GTSE1 was shown to be a regulated cytoskeletal protein required to promote cell migration [25]. Previous studies have also reported that GTSE1 was identified as a novel YAP/TAZ-TEAD4 regulatory protein that could promote metastasis in breast cancer cells when it is overexpressed, especially in TNBC [26, 27]. However, up to now, there has been no detailed study regarding the functional role of GTSE1 in breast cancer.

In the present study, we showed that GTSE1 was associated with worse outcome and malignant phenotype including enhanced abilities of tumor proliferation and metastasis in breast cancer. Moreover, GTSE1 contributed to multidrug resistance in breast cancer cells. Intriguingly, GTSE1 could regulate the p53 function to alter the cell cycle distribution, dependent on the mutational status of p53. Taken together, we brought light on the function of GTSE1 and showed its potential significance as a novel biomarker for assessing the breast cancer progression.

## Methods

### TCGA data analysis and bioinformatics analysis

*RNA-Seq* data of normal breast and breast cancer tissues were downloaded from TCGA and analyzed to find genes that were significantly upregulated in breast tumors by using the EdgeR method. The candidate genes were identified by the following conditions: (1) the genes had to be significantly upregulated in samples of breast cancer as compared to samples from normal breast tissue, (false discovery rate [FDR] < 5%); (2) the expression difference should be at least two of fold change; (3) the direction of gene expression had to be inversely and significantly associated with survival (*P* < 0.05). Survival analyses of the candidate genes were performed using the two online databases namely bc-GenExMiner and PROGgeneV2, to determine the prognostic significance of the mRNA expression level of GTSE1, and to perform further bioinformatics analysis.

### Cell lines and cell culture

The HCC38, MDA-MB-468, MDA-MB-231, MCF7, MCF10A cells were purchased from the American Type Culture Collection (ATCC). HCC38 cells were cultured in RPMI 1640 medium (GIBCO, C11875500BT) supplemented with 10% fetal bovine serum (FBS). MCF10A cells were cultured in RPMI 1640 medium supplemented with horse serum (5%), insulin (10μg/mL) and epidermal growth factor (20 ng/mL), hydrocortisone (0.5μg/mL). MCF7, MDA-MB-231 and MDA-MB-468 cells were grown in Dulbecco’s modified Eagle’s medium (GIBCO, C11995500BT) supplemented with 10% FBS at 37 °C and 5% CO2,100 units per mL penicillin (MDbio, P003-10 g), and 100 mg/mL streptomycin (MDbio, S007-25 g).

### Quantitative real-time polymerase chain reaction

Trizol reagent (Invitrogen) was used to extract total RNA from cultured cell lines, which was then applied to reverse transcription using a cDNA Synthesis Kit (ThermoFisher, EP0441). qRT PCR was conducted using a UNICONTM qPCR SYBR® Green Master Mix (YEASEN, 11198ES08). Expression data were standardized to the reference gene GAPDH in order to control the variability in expression levels and calculated as 2^- [(CT of candidate genes) - (CT of GAPDH)]^, where CT represents the threshold cycle for each transcript. The average for each gene and sample was calculated and the experiments were independently repeated three times. The primers used are listed in the Additional file 1: Table S1.

### Western blotting

Western blotting was performed according to the standard protocol. The primary antibodies, including GTSE1(Proteintech, 21,319–1-AP), Ki67(Proteintech, 27,309–1-AP),mutant p53(Abcam, ab32049), p53(Proteintech, 10,442–1-AP),Cyclin D1(Proteintech, 60,186–1-Ig),Cyclin E1(Proteintech, 11,554–1-AP), p21(Proteintech,60,214–1-Ig), E-Cadherin (Proteintech, 20,874–1-AP), Desmoplakin (Proteintech,25,318–1-AP), N-Cadherin (Proteintech,22,018–1-AP), Vimentin (Proteintech, 10,366–1-AP), Nanog (Proteintech, 14,295–1-AP), ABCG2(CST, 42078S), TAZ (CST, 4883S), YAP (CST, 14074S), ERK1/2(Affinity, AF0155), phospho-ERK1/2(Affinity, AF1015), AKT (CST, 2938S), Phospho-Akt (Thr308) (CST, 13038S), Phospho-Akt (Ser473) (CST, X4060S), FoxC2(CST, 12974S),Slug (CST, 9585 T),Twist1(CST, 46702S),Snail (Proteintech, MG-3879 T) and GAPDH (Proteintech, 60,004–1-Ig) were used at a dilution of 1:1000.

### Clinical materials

We obtained 16 non-cancerous breast tissues and 37 primary breast cancer tissues from the Department of Breast Carcinoma, at the Sun Yat-sen University Cancer Center (Guangzhou, P. R China) to investigate the mRNA expression levels of GTSE1. A total of 154 paraffin-embedded primary breast cancer tissues were obtained from patients with informed consent who underwent surgery for primary breast cancer at the Sun Yat-Sen University Cancer Center between 2003.04 and 2011.12, with the approval of the Institutional Clinical Ethics Review Board of Sun Yat-Sen University Cancer Center (IRB Approval Number GZR2019–101). The immuno-histochemical (IHC) score was composed of a score for the percentage of positive tumor cells and the staining intensity grade. The percentage of positive cells were classified as follows: 0–5% of stained cells = 0, 6–25% = 1, 26–50% = 2, 50–75% = 3 and 76–100% = 4. The intensity score of positive cells contained the cytoplasmic and nucleus staining intensity. The staining intensity was classified into the following four grades: no staining = 0, weak staining = 1, moderate staining = 2 and strong staining = 3. Then multiplied positive proportion with intensity to produce a total score which varied from 0 to 12. We then used the follow-up data of 154 breast cancer cases (TNBC, *n* = 90, non-TNBC, *n* = 64) for further survival analysis after immunohistochemical (IHC) staining for GTSE1.

### Small interfering RNA transfection

siRNAs and the negative control small interfering RNA (NC) were purchased from GenePharma (sequences shown in Additional file 1: Table S2). Transient transfections were performed in an antibiotic-free medium using the Lipofectamine RNAiMAX Reagent (Invitrogen, 13,778,150) following the manufacturer’s protocol. The GTSE1 silencing was performed by transfecting the siRNAs in MDA-MB-231 and MDA-MB-468 cell lines for 48 h.

### Lentivirus transfection and transduction

To establish GTSE1 knockdown stable cell lines, the human GTSE1 ‘SureSilencing shRNA’ plasmids were purchased from GeneCopoeia (sequences shown in Additional file 1: Table S3). Lentiviruses were produced by co-transfecting 293 T cells with the GTSE1 shRNA or negative control shRNA plasmid along with packaging plasmids using Lenti-Pac HIV Expression Packaging Kit (GeneCopoeia, LT003). After transfection for 48 h, infectious lentivirus was harvested through a 0.45-filter (Millipore, SG079) before using them to infect the MDA-MB-231 cells. Then the infected cells were screened with 1 μg/mL of puromycin (MPbio, 219,453,925) for 7 days. To produce MDA-MB-231 and MCF7 cells with stable overexpression of GTSE1(sequences shown in Additional file 1: Table S4), the full-length human GTSE1 was cloned into a lentiviral vector. The efficiency of the knockdown and overexpression of GTSE1 was determined by Immunoblotting.

### MTS assay, and colony formation assay

Cell growth curves were plotted using the GraphPad software based on the data obtained from the MTS assays, which used the Cell Titer 96 Aqueous One Solution Cell Proliferation Assay kit (Promega, G3581) to detect the cancer cells viability and growth. In brief, a concentration of 1000 cells/200 μL of mixture medium was seeded into a 96-well plate (jet, TCP011096) and cultured under normal conditions for 7 days and tested MTS every day. At each corresponding time points after seeding, we incubated cells with 200 μL of the mixture of MTS and DMEM (1:10) for 2.5 h, and adjusted the absorbance to 490 nm to detect its viability with a microplate research reader (Bio-Tek EPOCH2, America). In order to perform the colony formation assay, 1000 cells/2 mL were seeded into a 6-well plate (jet, TCP011006) and the cultured medium was changed twice a week. Two weeks later, the colony number were calculated after washing these cells with 1X phosphate-buffered saline (MP, 92810307) solution, fixing them with methanol for 20 min, and staining with 2% crystal violet for 30 min. These experiments were independently repeated three times.

### Analysis of cell motility

Cell motility was determined by using the transwell and wound healing assays. Migration assays were performed using chambers without Matrigel (Falcon, 353,097), and invasion assays were conducted with the Matrigel Invasion Chamber (Corning, 354,480) based on the manufacturer’s protocol. Briefly, cells in 200 μL of serum free medium were seeded to the upper chamber and allowed to migrate or invade to the other side of the membrane. After 48 h, the chambers were stained with crystal violet. Images were captured from each membrane using the ImageJ software to count for the metastatic cell numbers. In migration assay, 30,000 number of cells were seeded to the upper chamber and the chamber was not covered with Matrigel. While in invasion assay, 50,000 number of cells were seeded to the upper chamber and the chamber was covered with Matrigel. After 48 h, the chambers of transient transfection groups (siNC and siGTSE1) were stained with crystal violet, and the stable cell line groups (vector and GTSE1) were stained after 30 h.The mean number obtained from three independent assays under each experimental condition was used in the final analysis. The wound healing assay was conducted using a 200 uL pipette tip to scratch the cell layer 24 h after seeding, and the wound healing rate was recorded after 0, 24, 36, 48 and 72 h. All experiments were independently repeated three times.

### Sphere culture assay

A concentration of 1000 cells/well were seeded on 6-well low-attachment culture plates (Corning, 3471) in order to perform the tumor sphere assay. Cells were cultured in serum-free DMEM/F12 medium (GIBCO, C11330500BT) supplemented with 20 ng/ml of EGF (epidermal growth factor) (NovoProtein, C029-B) and 20 ng/mL of bFGF (basic fibroblast growth factor) (NovoProtein, C046-A). The number of spheres was counted using the Image J software. Three independent experiments were performed.

### Cell apoptosis and cell cycle assay

Cell apoptosis and cell cycle were respectively detected by the Annexin V FITC Apop Dtec Kit (BD, 556547) and a cell cycle staining kit (BestBio, BB-4104-3), in accordance to the manufacturer’s manual. Briefly, the cells were grown to 70% confluency, after 48 h of incubation with or without drug (Taxol, 5-fluorouracil, and Adriamycin), the cells were collected and processed for analysis. The cell samples were then analyzed using a flow cytometer ACEA NovoCyte equipped with the FlowJo software (version 0.7).

### Xenograft tumor model

All animal experiments were conducted according to the instructions approved by the Research Animal Resource Center of Sun Yat-Sen University (Approval number L102042018030E). Three-week-old female BALB/c mice were purchased from the Sun Yat-sen University Laboratory Animal Center and maintained under standard conditions. Tumor cells of MDA-MB-231 scramble and shGTSE1 groups (2 × 10^6^ cells/tumor in 100 μl DMEM) were suspended in 100 μl DMEM containing 50% Matrigel (Corning, 356,237) and injected into their left and right axillary areas. The mice were observed three times per week for apparent tumor formation. The experiment was finished at 22 days after tumor cell inoculation. For lung metastasis experiment, the tumor cells of MDA-MB-231 scramble and shGTSE1 groups (1 × 10^6^ cells/tumor in 100 μl DMEM) were intravenously injected through the tail vein of the mice. Lung metastatic nodules were calculated after 5 weeks when the mice died of cachexia. Lungs were excised for hematoxylin and eosin staining. In addition, six-week-old female BALB/c mice were used to perform situ breast pad injection. Tumor cells of MCF7 vector and GTSE1-overexpression groups (7 × 10^6^ cells/tumor in 100 μl DMEM) were suspended in 100 μl DMEM containing 50% Matrigel and injected into the left and right breast pad areas. The mice received estradiol supplementation (0.4 mg/kg) every 7 days for 35 days after cell injection, and were observed and palpated for tumor appearance. Tumor growth was measured weekly using calipers. Tumor volume was determined using the standard formula: L*W^2^*0.52, where L and W refered to the longest and shortest diameters, respectively.

### Senescence-associated β-gal assay

Senescence-associated β-gal assay was carried out to determine the cells’ senescence state using an SA-β-gal staining kit (Roche, 11,828,673,001) according to introductions. Briefly, the cells were washed with PBS (1X), fixed with 2% formaldehyde and 0.2% glutaraldehyde for 10 min after removing medium, the cells were washed with PBS twice and incubated with fresh SA-β-gal stain solution, which was then kept overnight at 37 °C without CO2. The cells were then photographed using a microscope (Nikon Eclipse 80i), and the percentage of senescence cell was counted by counting three random fields.

### Statistical analyses

Statistical analyses were conducted using the GraphPad Prism version 7.0 (USA, GraphPad Software) and the SPSS software version 22.0 for Windows (IBM, USA). The relationship between GTSE1 expression and clinicopathologic status was analyzed using the chi-square test. The correlations between the GTSE1 expression to OS and RFS were analyzed using the Kaplan–Meier survival and log-rank test. Data were analyzed using the Student’s t-test or ANOVA methods. *P*-value of less than 0.05 was considered as statistically significant for all tests.

## Results

### Identification of GTSE1 in breast cancer progression based on the analysis of the online databases

**Fig. 1:**
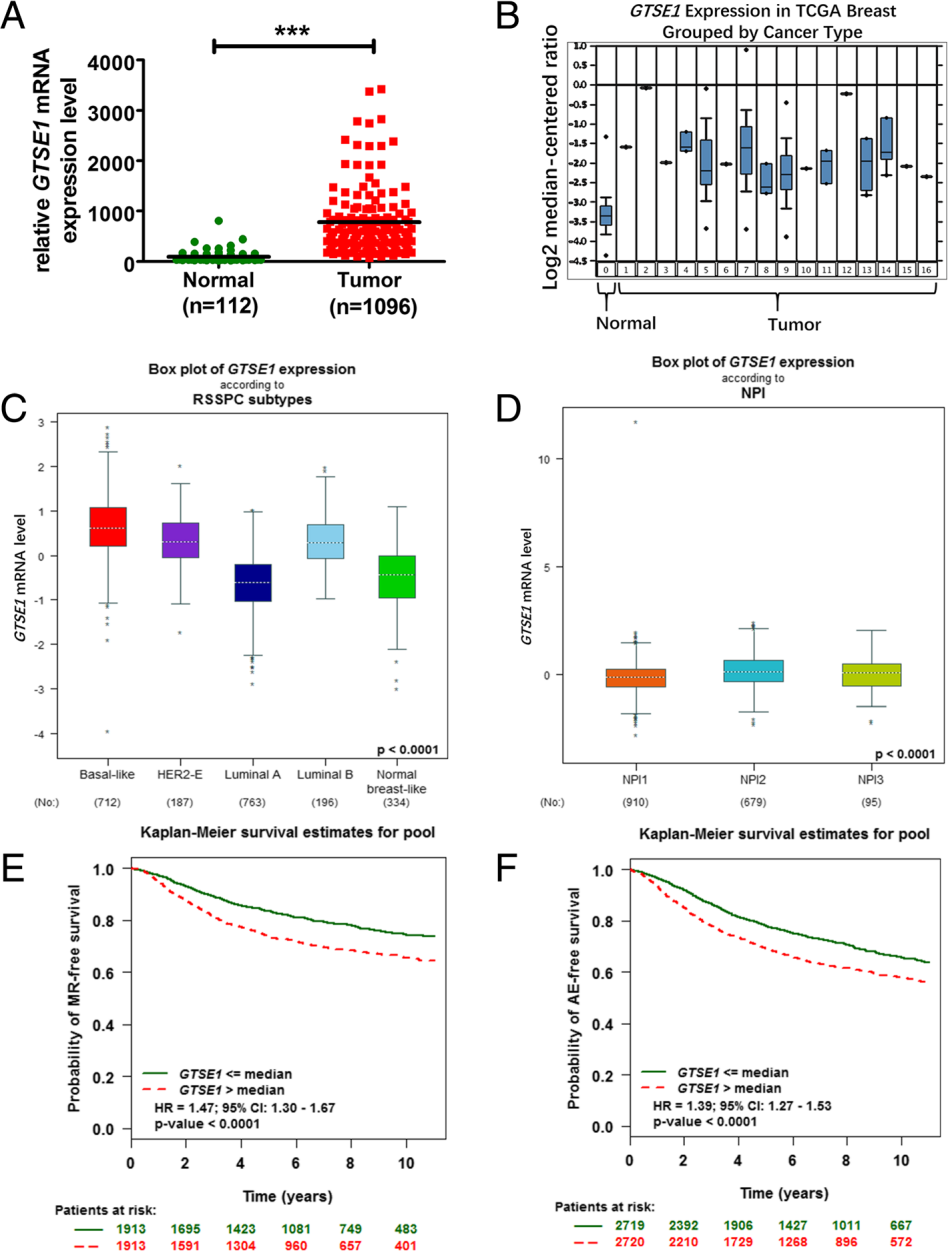
Identification of GTSE1 in breast cancer progression based on database. **a** Expression level of GTSE1 was elevated in 1096 breast cancer tissues compared with 112 normal breast tissue samples in the TCGA profile. **b** GTSE1 expression was significantly upregulated in different breast cancer pathological types in TCGA profile based on the Oncomine (**c, d, e** and **f**) and bc-GenExMiner v4.1 databases. **c** GTSE1 expression was positively correlated with the degree of malignancy of different breast cancer subtypes. **d** GTSE1 expression was positively correlated with the Nottingham Prognostic Index (NPI) of breast cancer. **e** Metastatic relapse-free survival for patients with high or low GTSE1 mRNA expression. *n* = 3826, *p* < 0.0001, HR = 1.47. **f** GTSE1 low expression had a significantly better survival rate than that of high-expression patients. *n* = 5439, *p* < 0.0001, HR = 1.39

*RNA-Seq* data and other associated survival data of breast cancers as well as normal breast tissues were downloaded from the TCGA database for further analysis in order to identify genes crucial for breast cancer progression. In this study, we chose to focus on GTSE1 for the following three determinant causes: it is up-regulated in breast cancer tissues according to TCGA database (Fig. 1a), and the results from the Oncomine database indicated that compared with normal breast tissue, its expression was higher in different kinds of breast cancer pathological types (Fig. 1b); its expression was positively correlated with the degree of malignancy of different breast cancer subtypes (Fig. 1c); the higher its expression, the higher the Nottingham prognostic index, the worse the prognosis of breast cancer (Fig. 1d) (NPI, the Nottingham prognostic index is used to assess the prognosis after breast cancer surgery, which includes three pathological criteria: lesion size; the number of lymph nodes involved; and the tumor grade) [28]; and the expression is inversely correlated with metastatic relapse-free survival (Fig. 1e) and any event-free survival (Fig. 1f) according to bc-GenExMiner v4.1 database [29].

### p53 mutation is correlated with the high expression of GTSE1

**Fig. 2:**
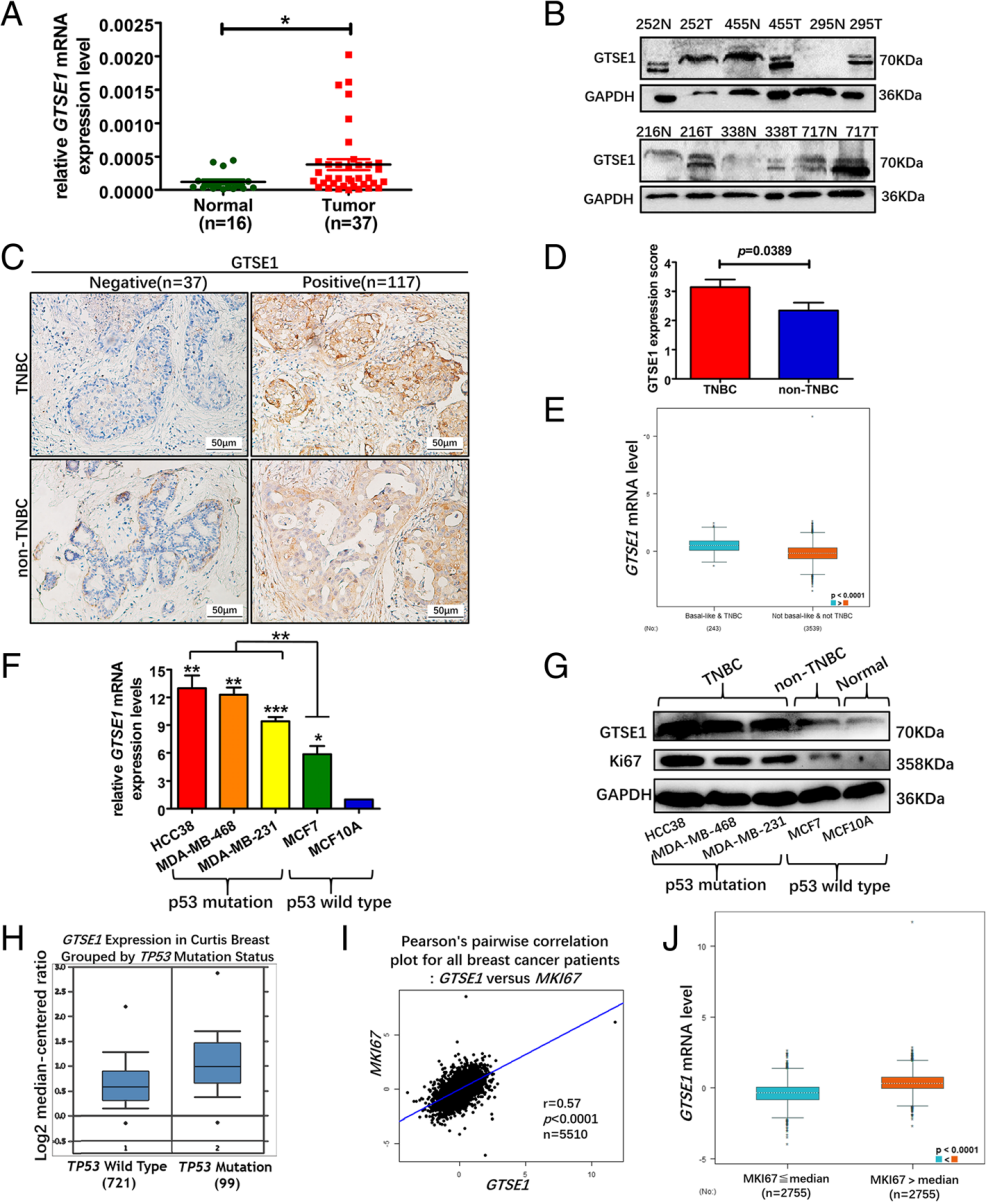
P53 mutation is correlated with the high expression of GTSE1. **a** GTSE1 mRNA expression in the breast cancer tissues and normal breast tissues detected by qPCR. GTSE1 mRNA expression level was standardized to the expression level of housekeeping gene GAPDH. **b** GTSE1 protein expression level in six paired breast tumor tissues (T) and their adjacent normal tissues (N). **c** Breast cancer tissues containing 64 non-TNBC and 90 TNBC were stained with GTSE1 antibodies. Representative images of immunohistochemistry (IHC) staining were shown. **d** GTSE1 protein expression level was higher in TNBC compared to non-TNBC. **e** GTSE1 mRNA expression level was higher in TNBC compared to non-TNBC based on the bc-GenExMiner v4.1 database. **f** Real-time PCR analysis of GTSE1 mRNA expression level in normal breast epithelium cell line MCF10A and breast cancer cell lines. **g** Western blotting of GTSE1 and Ki67 expression in normal breast epithelium cell and breast cancer cell lines. **h** Based on Oncomine database, the expression level of GTSE1 was higher when p53 was mutated than that of wild type p53. **i** and **j** Based on the bc-GenExMiner v4.1 database, the GTSE1 mRNA expression level was positively correlated with MKI67. *r* = 0.57, *p* < 0.0001, *n* = 5510. GAPDH was tested as a loading control in the immunoblotting. Repeating the experiment three times independently

GTSE1 mRNA expression level (Fig. 2a) and the GTSE1 protein level (Fig. 2b) was higher in the breast cancer tissues as compared to the normal breast tissues. Immunohistochemistry staining showed that GTSE1 was mainly located in the cytoplasm of breast cancer cells (Fig. 2c), and its protein expression level was higher in TNBC (Fig. 2d), which was consistent with the result of the bc-GenExMiner database showing the GTSE1 mRNA level (Fig. 2e). Quantitative real-time PCR and western blotting of GTSE1 showed that it was highly expressed at various levels in different breast cancer cell lines especially in TNBC. Since GTSE1 was the target gene of p53 [14], the expression level of GTSE1 was higher in the p53 mutated cell lines than that of wild type p53 cell line (Fig. 2f and g), and these results were confirmed by the results obtained from the Oncomine database (Fig. 2h). The wild-type p53 gene is an important anti-oncogene, and mutant p53 gene coding protein losses its tumor suppressive functions and can induce malignant transformation of cells [30, 31]. MCF7 is a luminal epithelial breast cancer cell line and its malignancy is lower than that of triple negative breast cancer cell lines such as HCC38, MDA-MB-231 and MDA-MB-468 cells [32, 33]. p53 is mutated in almost all TNBC [34]. Since GTSE1 is one of the target genes of p53, we discovered that the expression level of GTSE1 was lower in wild type p53 cell lines such as MCF7 and normal breast epithelial cell line MCF10A compared to p53 mutated cell line such as HCC38, MDA-MB-231, and MDA-MB-468 cells. These results suggested that increased GTSE1 expression may have been due to the mutation of the transcription factor p53, which results in the loss of tumor suppressive function. Moreover, the GTSE1 mRNA expression level is positively correlated with MKI67, which was consistent with the results of the protein level (Fig. 2g, i and j), suggesting that GTSE1 may be a potential tumor marker for prognosis. However, previous study showed that Ki-67 is a controversial biomarker in breast cancer. Immunohistochemical detection of Ki-67 in breast cancer is an important indicator of molecular classification of breast cancer, and the Ki-67 index is closely related to individualized treatment and prognosis of breast cancer. However, the interpretation repeatability of Ki-67 immunohistochemical test results by different observers was poor. So far, there is no standardized method and corresponding critical value of Ki-67 immunohistochemical interpretation of breast cancer [35, 36].

### GTSE1 is closely related to differentiation and prognosis of breast cancer

**Fig. 3:**
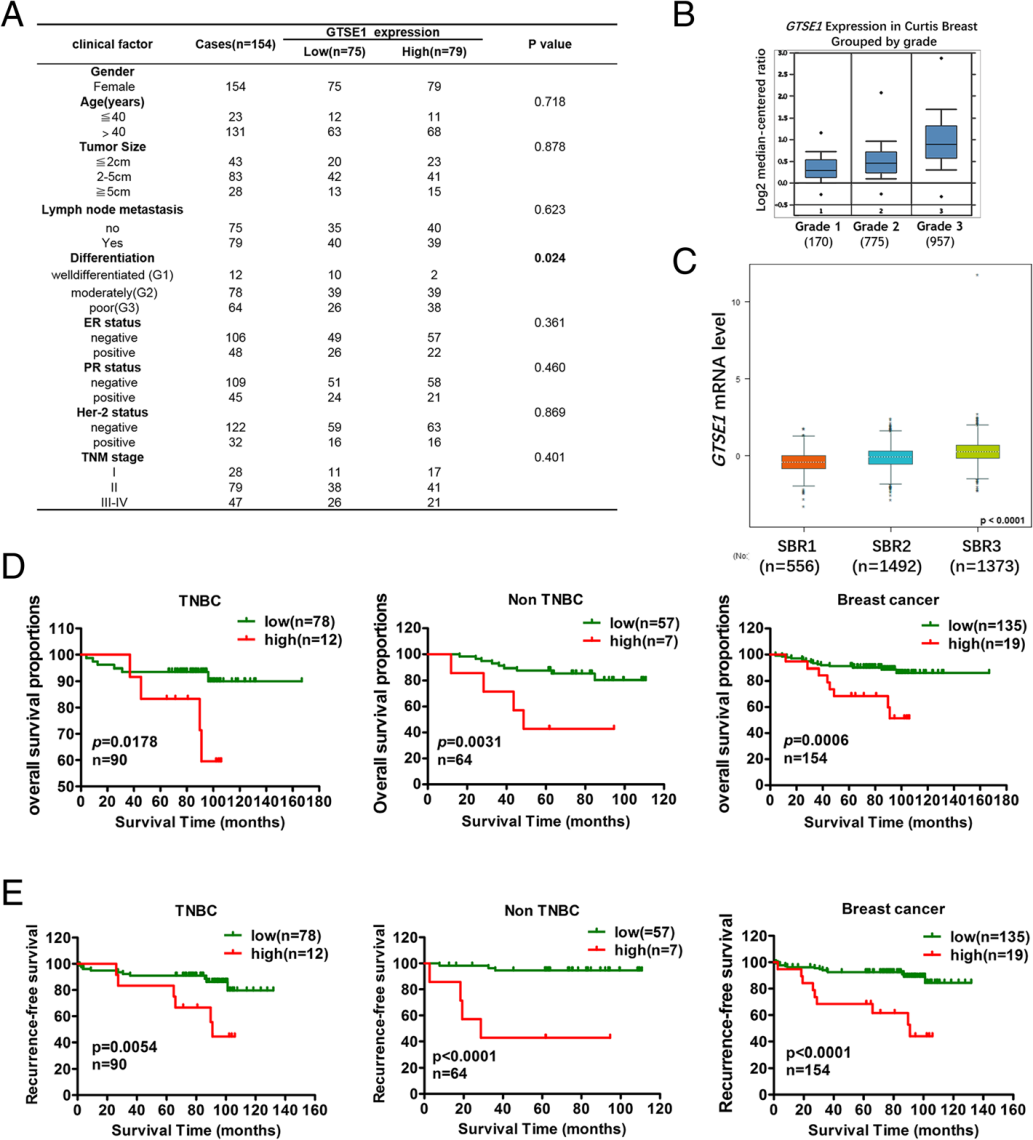
GTSE1 is closely related to the differentiation and prognosis of breast cancer. **a** GTSE1 expression in primary tumors were significantly related to a higher histological differentiation (*p* = 0.0024). **b** Oncomine database showed that the GTSE1 expression was positively correlated with histological grade. **c** Bc-GenExMiner database showed that GTSE1 expression was positively correlated with Scarff-Bloom-Richardson (SBR) histological classification. **d** and **e** Kaplan–Meier curve was used to analyze the protein expression data from IHC by using GraphPad software. GTSE1 high expression levels were negatively correlated with overall survival (OS) and recurrence-free survival (RFS) in TNBC and non-TNBC patients

The GTSE1 protein expression level with clinical and pathological characteristics in 154 breast cancer (cases including TNBC and non-TNBC were attached in Additional file 2: Table S5 and Additional file 3: Table S6 respectively) was analyzed, and these samples were divided into two groups by the median value of immunohistochemical scores as the cut-off value. We performed the χ2 test, which demonstrated that the increased GTSE1 expression in primary breast tumors were significantly related to a higher histological grade (*p* = 0.0024) (Fig. 3a). The Oncomine and bc-GenExMiner database showed that the GTSE1 expression was positively correlated with histological grade (Fig. 3b) and the Scarff-Bloom-Richardson (SBR) histological classification (Fig. 3c). Scarff-Bloom-Richardson has been widely used in clinical practice, which is conducted through comprehensive evaluation, on a hematoxylin eosin (H&E) staining, tubular formation of tumor, nuclear pleomorphism and mitotic count [37]. Tumor grade of differentiation of breast cancer is a definitive prognostic factor. The grade is usually reported on a scale ranging from 1 to 3, including well differentiated Grade 1 (G1), moderately differentiated Grade 2 (G2) and poorly differentiated Grade 3 (G3), where G3 tumors are the most aggressive [38]. Kaplan–Meier survival curves using optimum cut-off value revealed that GTSE1 high expression levels were negatively correlated with overall survival (OS) and recurrence-free survival (RFS) in TNBC and non-TNBC patients (Fig. 3d and e), which was consistent with the survival data from the NNK, GSE6532, and GSE11121 datasets in the PROGgeneV2 database [39] (Additional file 4: Figure S1A, B and C). Collectively, these data showed that the expression level of GTSE1 was closely related to the poor prognosis of these patients.

### GTSE1 promotes breast cancer cell growth by activating AKT pathway

**Fig. 4:**
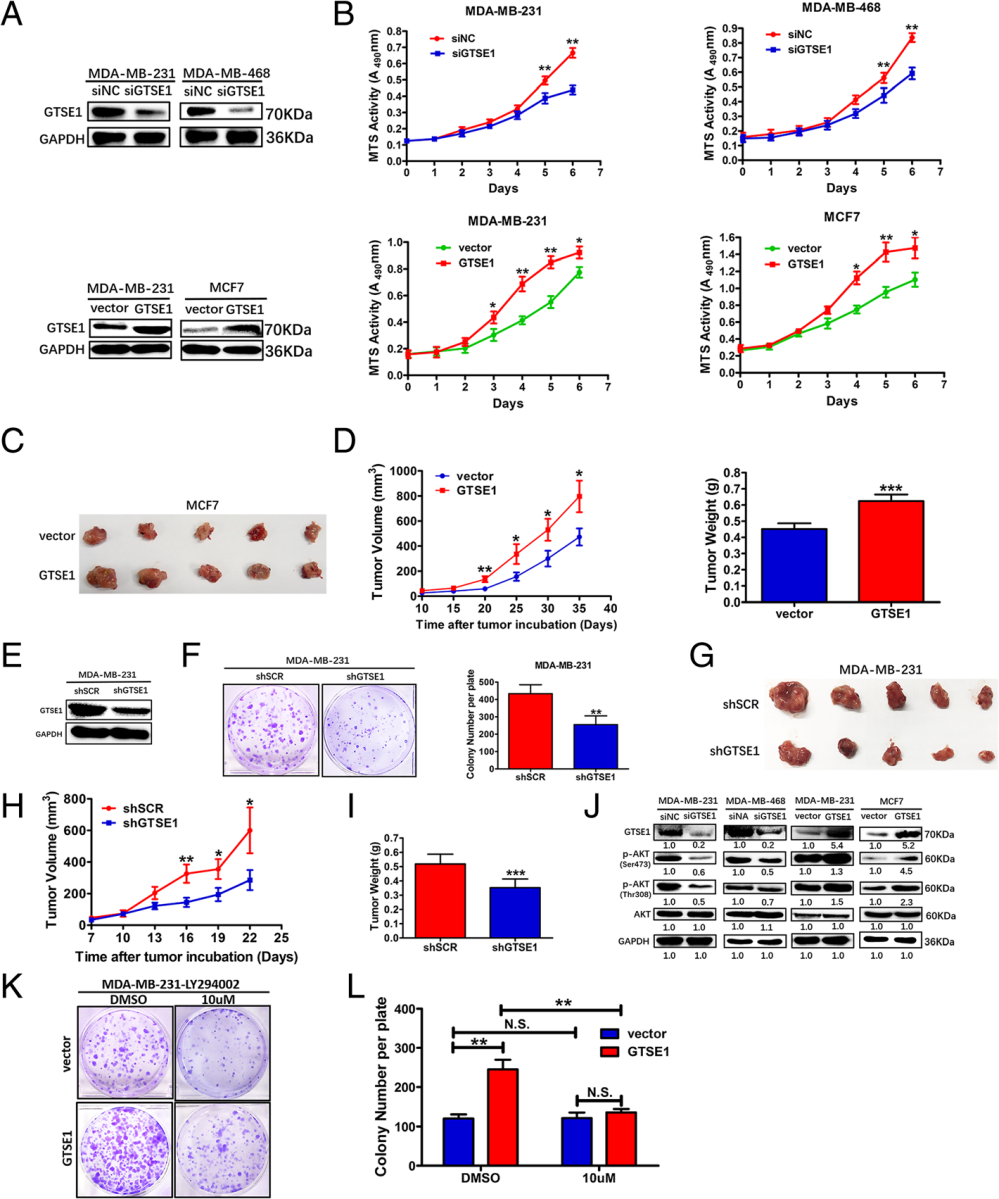
GTSE1 promoted breast cancer cell growth by activating AKT pathway. **a** Decreased expressions of GTSE1 were confirmed by Western blotting in GTSE1-silenced MDA-MB-231 and MDA-MB-468 cells compared with negative control siRNA cells, and GTSE1 overexpression level in MDA-MB-231 and MCF7 cells was validated by immunoblotting. **b** Cell growth rates were measured by the MTS assay. **c** Tumor masses isolated from MCF7 stably overexpressing GTSE1 or vector after tumors had grown for 5 weeks. **d** Overexpression of GTSE1 significantly promoted xenograft tumor volume in female nude mice the final tumor weights. *p < 0.05, **p < 0.01, ***p < 0.001, Student’s t-test. The data are presented as the mean ± S.D. (*n* = 5 per group) **e** Stable decreased expression of GTSE1 was confirmed by immunoblotting in GTSE1 silenced MDA-MB-231 cells compared with scrambled shRNA control cells. **f** The colony formation assays were performed in GTSE1 stably knockdown and control cells to detect the effect of growth. The data are presented as the mean ± S.D. **p < 0.01, Student’s t-test. (from triplicates). **g** Tumor masses collected from MDA-MB-231 stably expressing Ctr or GTSE1 shRNA after tumors had grown for 22 days. **h** Knockdown of GTSE1 significantly reduced xenograft tumor growth and volume in female nude mice. **i** Compared to the negative control shRNA, the final tumor weights were decreased significantly. ***p < 0.001, Student’s t-test. The data are presented as the mean ± S.D. (*n* = 5 per group). **j** Phosphorylated and total AKT level in human breast cancer MDA-MB-231 and MDA-MB-468 cells silencing GTSE1 or negative control siRNA, and MDA-MB-231 and MCF7 cells stably expressing a control vector or GTSE1 were determined by immunoblotting. **k** and **l** Plate clone formation assays were conducted in the indicated cell lines after a specific AKT inhibitor LY294002 treatment

To investigate the role of GTSE1 in breast cancer cell proliferation, we transfected MDA-MB-231 and MDA-MB-468 cells with siRNA (GTSE1 si#1) and negative control siRNA. Since our qPCR results showed that the MDA-MB-231 cell line had a moderate expression level of GTSE1, we performed both gain and loss of function in MDA-MB-231. We established GTSE1 overexpression cell lines in MDA-MB-231 and MCF7. The GTSE1 protein expression level in these transformed cells was confirmed by western blotting (Fig. 4a). Silencing the GTSE1 significantly suppressed the MDA-MB-231 and MDA-MB-468 cells proliferation, as well as their colony forming ability, while the overexpression of GTSE1 in both MDA-MB-231 and MCF7 cells promoted the cellular proliferation and colony formation ability (Additional file 5: Figure S2A and B). To sum up, these results demonstrate that GTSE1 could promote breast cancer cell growth in vitro. We established the MDA-MB-231 cell line with GTSE1 stably silenced via lentiviral infection and validated by immunoblotting (Fig. 4e). Colony formation assay showed that the knockdown of GTSE1 obviously decreased the colony numbers and size compared with the SCR group (Fig. 4f). Then, we performed an animal xenograft model using the MDA-MB-231 cell line stably silenced GTSE1 or shSCR, and MCF7 cell line stably overexpressed GTSE1 or vector. The pictures of the isolated tumors are shown (Fig. 4g, Fig. 4c), the tumor growth and weight were found to be reduced in the MDA-MB-231-shGTSE1 cell group as compared with the control group and increased in the MCF7-GTSE1 cell group as compared with the vector group (Fig. 4h and i; Fig. 4d). Since the AKT pathway has a crucial influence on breast cancer malignant phenotype [40, 41], we determined that GTSE1 had an effect on AKT activation. Immunoblotting showed that GTSE1 level was positively correlated with the phosphorylated AKT. Ectopic expression of GTSE1 increased the phosphorylated AKT levels without affecting the total AKT levels in breast cancer cells (Fig. 4j). After a specific AKT inhibitor LY294002 treatment, MDA-MB-231 cells stably overexpressing GTSE1 were subjected to colony formation assays, and we observed that LY294002 could reverse their proliferation abilities (Fig. 4k and l). These suggest that GTSE1 could promote breast cancer growth at least partially by activating the AKT pathway.

### GTSE1 promoted breast cancer metastasis by regulating EMT

**Fig. 5:**
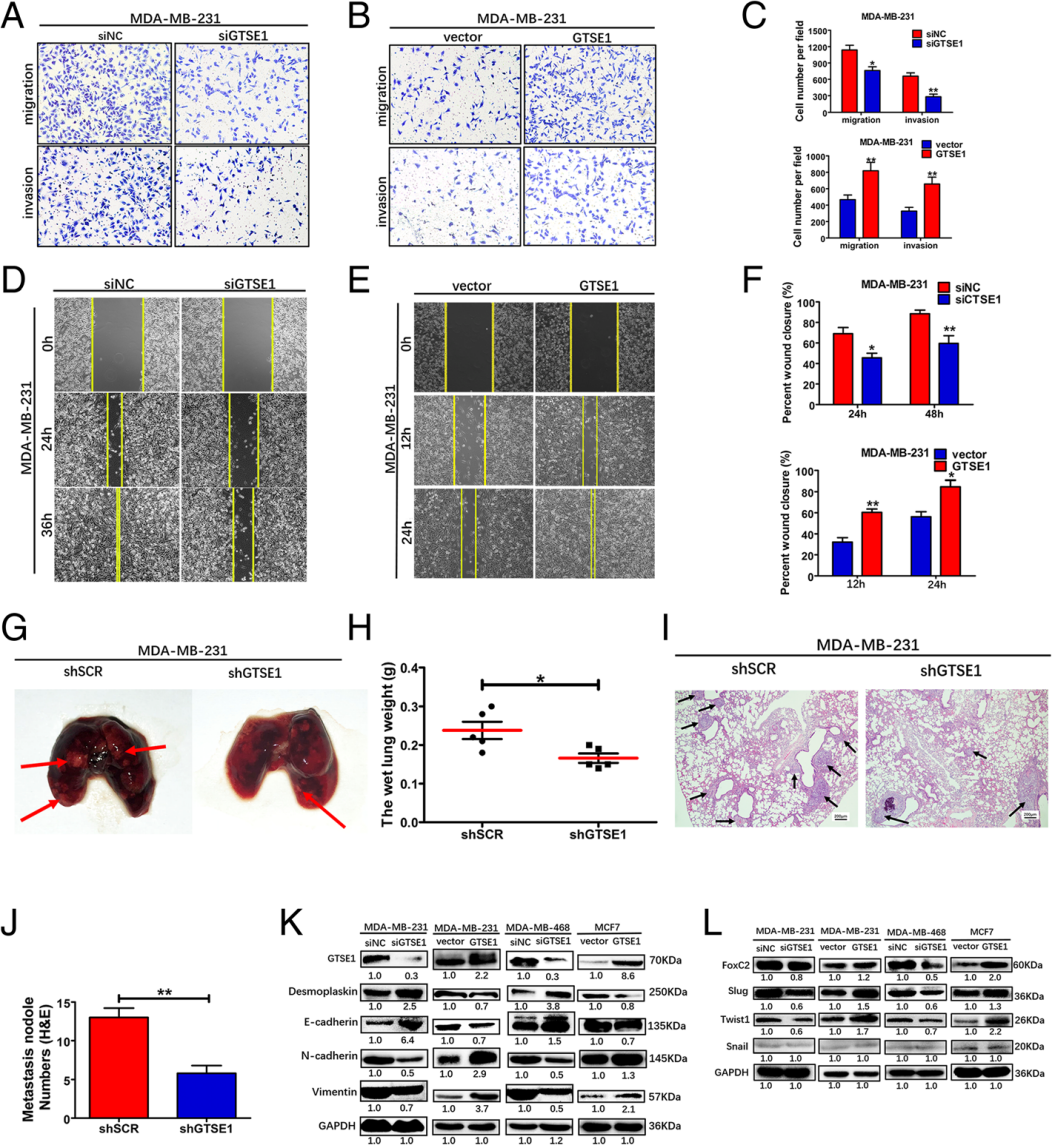
GTSE1 promotes breast cancer metastasis by regulating EMT. **a** and **b** Silencing GTSE1 could restrain the migratory and invasive ability in MDA-MB-231 cells compared with siRNA negative control cells. **d** and **e** Silencing or overexpression of GTSE1 distinctively changed cell migratory ability as detected by wound scratch assay. Yellow lines signify the wound margins. **c** and **f** Columns, average of three independent experiments; Bars, S.D. *p < 0.05, **p < 0.01. **g** Stably transfect-GTSE1 knocked-down cells (SCR, shGTSE1) were injected in mice via the tail vein, the lung of mice was washed by 0.9% NaCl. **h** The wet lung weight was recorded. **i** Lung sections were stained with hematoxylin and eosin. **j** The numbers of microscopic metastatic nodules in the sections were counted. Data were presented as the mean ± S. D, *n* = 5 per group. **k** The protein levels of EMT markers were detected by immunoblotting analyses in the indicated cells. **l** Western blot analysis of the regulators of EMT in indicated cells, and GAPDH as a loading control

To explore the biological function of GTSE1 in breast cancer metastasis, we performed transwell assay in MDA-MA-231 and MDA-MB-468 cells using the RNA interference (RNAi) to knockdown GTSE1 expression, which suggested that GTSE1 depletion significantly restrained the migration and invasion abilities of MDA-MB-231 cells (Fig. 5a and c). However, ectopic overexpression of GTSE1 had the opposite effect (Fig. 5b and c) and also improved the migration ability in MCF7 cells (Additional file 6: Figure S3A). Wound healing assay further demonstrated that GTSE1 silencing inhibited cell motility and the overexpression of GTSE1 promoted cell motility (Fig. 5d, e and f; Additional file 6: Figure S3B, C, D and E). To further confirm the metastasis ability promoted by GTSE1 in vivo, we established a metastasis model by injecting stably knocked down GTSE1 MDA-MB-231 cells into the tail vein of nude mice, and found that the knocked-down of GTSE1 significantly inhibited metastasis to the lungs. In addition, the number of metastatic nodules at the lung surface in the control group was significantly more frequent and larger in size than that in the knocked-down group (Fig. 5g). The lungs of the control group were heavier than that of the knockdown group (Fig. 5h). This phenomenon was further confirmed by examining the lung tissues using HE staining (Fig. 5i and j). A high level of GTSE1 accompanied reduced levels of epithelial protein such as Desmoplakin and E-cadherin and elevated levels of mesenchymal markers such as N-cadherin and vimentin in the GTSE1-overexpressing cells, and knockdown GTSE1 demonstrated reverse results (Fig. 5k). In addition, the western blot results of regulators of EMT including markers such as Snail, Slug, Twist1 and FOXC2 were also consistent with expectations (Fig. 5l) Collectively, these results demonstrated that GTSE1 could regulate the epithelial-mesenchymal transition. The survival data of GSE5327 based on the PROGgeneV2 database also showed that lung metastasis free survival was reduced in the GTSE1 high expression group (Additional file 6: Figure S3I), suggesting that GTSE1 is a metastatic promoting factor. Hence, we demonstrated that GTSE1 could promote breast cancer cells invasion and metastasis by regulating the epithelial-mesenchymal transition. LY294002 did not reverse the invasive phenotypes and EMT maker of MDA-MB-231 cells stably expressing GTSE1 (Additional file 5: Figure S2F, G and H). These results indicate that GTSE1 promotes metastasis independent of the activation of AKT signaling.

### GTSE1 can cause multidrug resistance in breast cancer cells

**Fig. 6:**
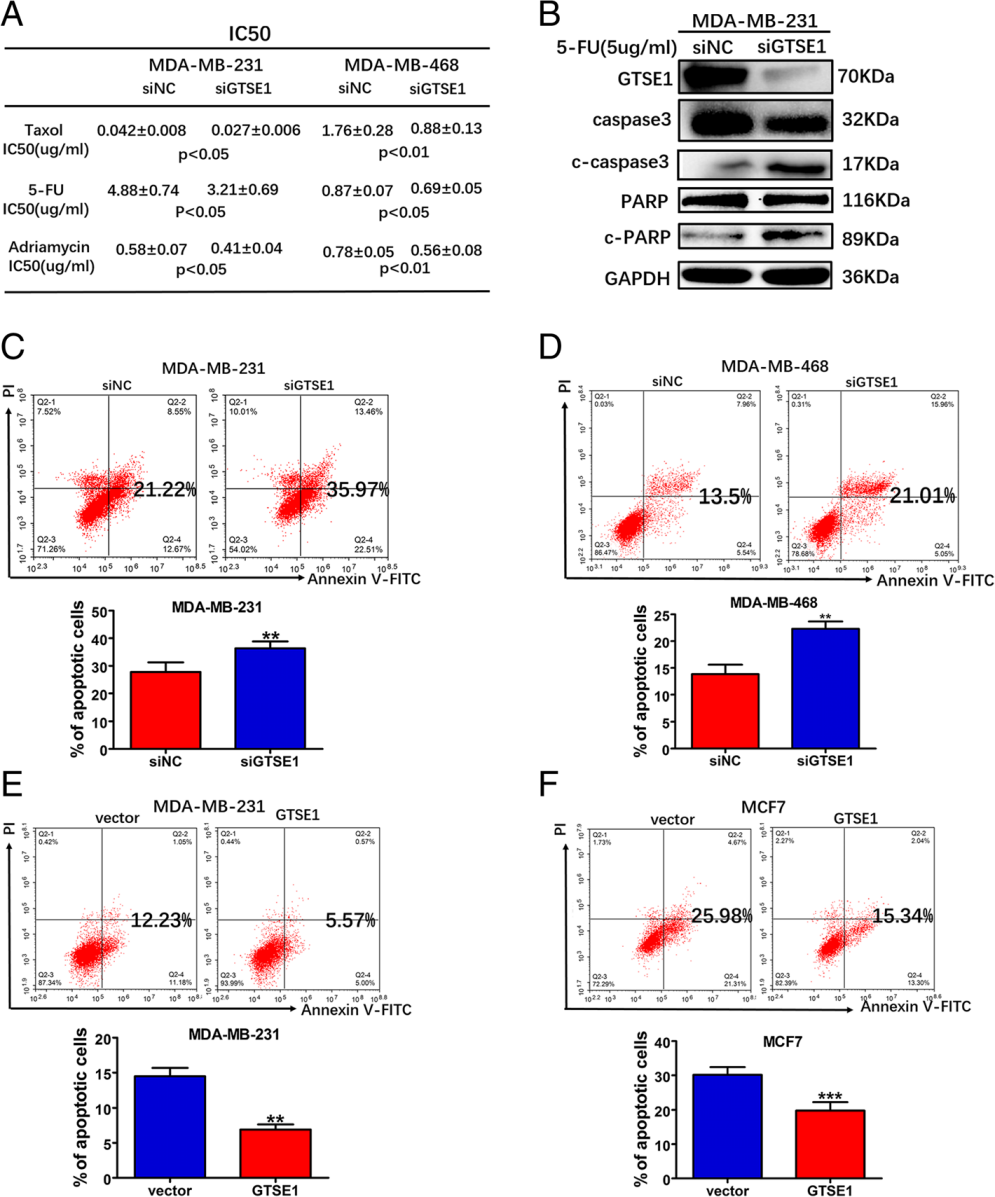
GTSE1 can cause multidrug resistance in breast cancer cells. **a** GTSE1 silencing MDA-MB-231 cells were treated with various concentrations of Taxol, 5-fluorouracil and Adriamycin for 72 h to detect the IC50 using MTS assays. **b** MDA-MB-231 transiently silenced GTSE and negative control cells were treated with 5-fluorouracil for 72 h, and the total PARP, cleaved PARP, total caspase-3 and cleaved-caspase-3 expression levels were determined by western blotting. GAPDH was tested as the loading control. **c** and **d** The indicated MDA-MB-231 and MDA-MB-468 cells were treated with 5-fluorouracil (5 and 0.9μg/ml respectively) for 48 h and test for apoptosis. **e** and **f** The indicated MDA-MB-231 and MCF7 cells were treated with 5-fluorouracil (5 and 20μg/ml respectively) for 48 h, then was tested for its apoptosis rate. Three independent experiments were repeated for each assay

Taxol, 5-fluorouracil and Adriamycin are commonly used chemotherapy drugs for TNBC patients [42–44]. To investigate the chemotherapy resistance function of GTSE1 in MDA-MB-231 and MDA-MB-468 cells, we determined the IC50 of Taxol, 5-fluorouracil and Adriamycin. We found that the knockdown of GTSE1 in MDA-MB-231 and MDA-MB-468 cells increased its sensitivity to Taxol, 5-fluorouracil and Adriamycin as compared with the control group (Fig. 6a). After 5-fluorouracil treatment, GTSE1-silencing cells had higher cleaved PARP and cleaved caspase-3 levels but had lower total caspase-3 levels than the control cells, which indicated that GTSE1 presents resistance to 5-fluorouracil (Fig. 6b). Furthermore, we performed FACS analysis to detect cell apoptosis, which showed that 5-fluorouracil induced cell apoptosis rates were increased in the MDA-MB-231 and MDA-MB-468 cells that transiently silenced GTSE1 as compared with the control cells (Fig. 6c and d). The cell apoptosis rates were reduced after overexpression of GTSE1 in MDA-MB-231 and MCF7 cells (Fig. 6e and f), the gating of these cells are explained in the supplemental data (Additional file 7: Figure S4A, B, C, D, E, F, G and H). β-Galactosidase assay showed that after 48 h of exposure to 5-fluorouracil, this caused alterations in MDA-MB-231 cells transiently silenced GTSE1, compared with the control cells, overall indicating senescence (Additional file 8: Figure S5A and B). These data emphasize the important function of GTSE1 in the multidrug resistance of breast cancer cells.

### GTSE1 regulates p53 function to alter cell cycle distribution dependent on the mutational status of p53

**Fig. 7:**
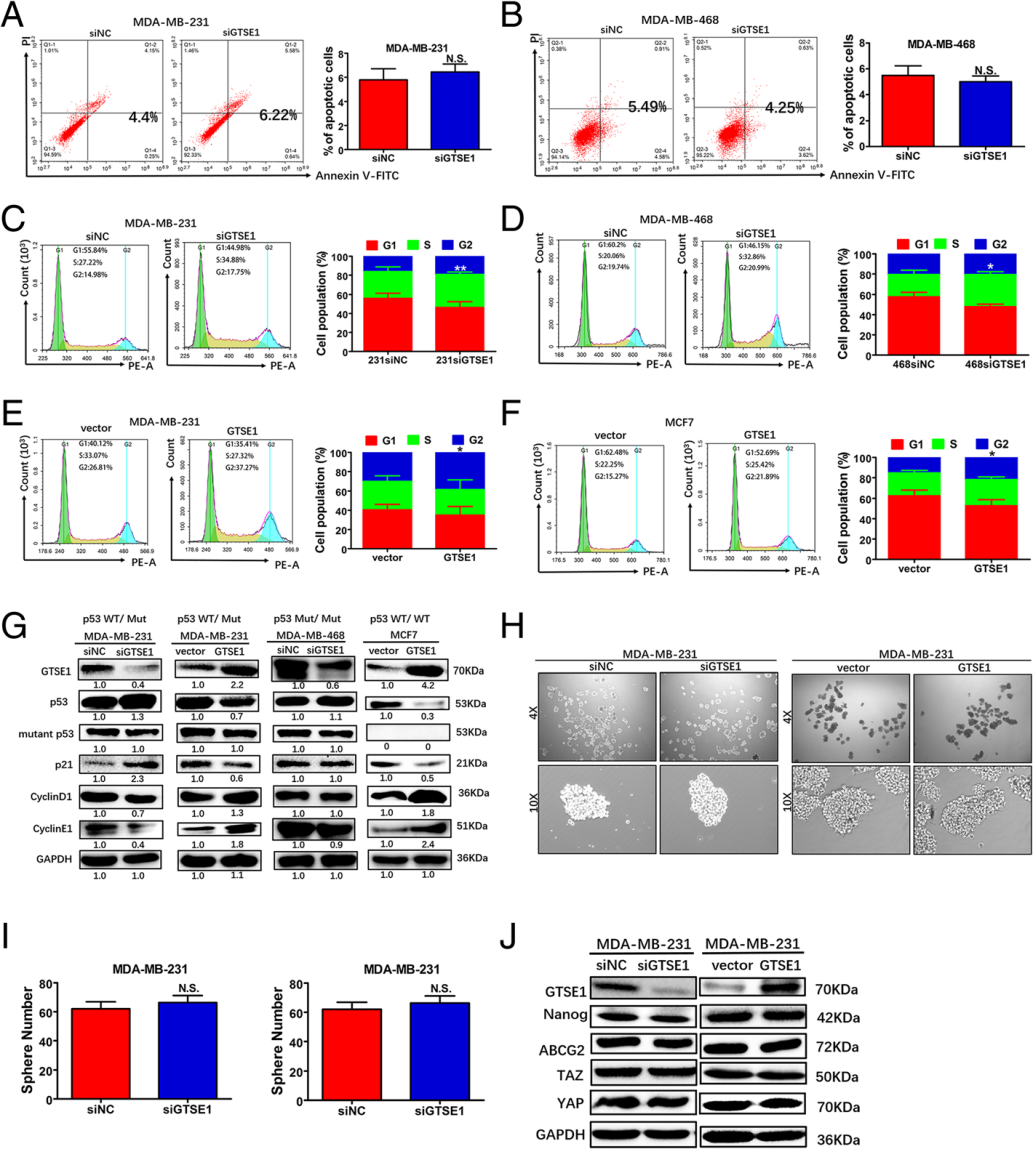
GTSE1 regulates p53 function to alter cell cycle distribution dependent on the mutation state of p53. **a** and **b** Indicated cells were collected without drug treatment for flow cytometry assays **c** and **d**. Downregulation of GTSE1 resulted in the accumulation of S phase cells by flow cytometry analysis. Indicated cells were stained with PI to analyze cell cycle distribution. Data are presented as means ±SD of three independent experiments. *p < 0.05, **p < 0.01, t-test. **e** and **f** Overexpression of GTSE1 results in accumulation of G2 phase cells by flow cytometry analysis. **g** Western blot analysis of GTSE1, P53, mutant p53, P21, cyclinD1, cyclinE1 expression in indicated cells. GAPDH served as the internal reference. Each experiment was repeated three times. **h** and **i** EGF and FGF were added to DMEM/F12 medium for 12–14 days to quantify the number and size of spheres formed by indicating cell lines. **j** Western blot analysis of Nanog, ABCG2, TAZ, YAP expression in indicated cells. GAPDH served as the internal reference

Flow cytometric analyses showed that there were no differences in cell apoptosis rates without 5-fluorouracil treatment (Fig. 7a and b), suggesting that the effect of GTSE1 on breast cancer cell proliferation was not via apoptosis. Moreover, flow cytometry was used to determine whether the role of GTSE1 in breast cancer cell proliferation affected cell cycle progression. Our data showed that silencing GTSE1 significantly increased S phase (Fig. 7c and d), suggesting that it may have an S phase arrest; while overexpression of GTSE1 increased the percentage of cells in the G2 peak (Fig. 7e and f), indicating that overexpression of GTSE1 could delay the M-to-G2 phase transition of breast cancer cells, which is consistent to a previous study. Moreover, immunoblotting assay showed that p53 and cell cycle inhibitor p21 were decreased in GTSE1- transfected MDA-MB-231 and MCF7 cells but was increased in GTSE1-silenced MDA-MB-231 cells. Conversely, cyclinD1 and cyclinE1 were upregulated in GTSE1-transfected MDA-MB-231 and MCF7 cells but downregulated in in GTSE1-silenced MDA-MB-231 cells. In addition, upregulation or downregulation of GTSE1 did not change p53, p21, cyclinD1 and cyclinE1 levels in GTSE1-silenced MDA-MB-468 cells. Compared with MDA-MB-468 cells with a mutant p53 homozygote, MCF7 cells with the wild-type p53 homozygote and MDA-MB-231 cells with a mutant p53 heterozygote exhibited a corresponding change in the level of p21 and cyclin protein (Fig. 7g), which indicated that GTSE1 had no effect on the mutant p53 homozygote but had an effect on the wild-type p53 homozygote and mutant p53 heterozygote. Taken together, these results reveal that GTSE1 could regulate the p53 function to alter cell cycle distribution dependent on the mutation state of p53 GTSE1. However, for the knockdown and overexpression of GTSE1 in MDA-MB-231 cells, the numbers and sizes of the spheres did not change (Fig. 7h and i) and the results were confirmed by western blotting (Fig. 7j). These results indicated that GTSE1 promotes the malignant phenotype of breast cancer without affecting the ability of tumor cells self-renewal.

## Discussion

Breast cancer is a heterogeneous malignancy, with multiple epigenetic and genetic alterations, and involving complex molecular signaling pathways [45–47]. In this study, we found that both the GTSE1 mRNA and protein expression levels are increased in breast cancer cell lines and tissues, especially in TNBC and p53 mutant breast cancer cells. Furthermore, its expression level was positively correlated with Ki67, highlighting its important role in human breast cancer progression. The expression level of GTSE1 protein in breast cancer tissues was related to differentiation and reduced overall survival time and recurrence free survival time of patients. Taken together, our study demonstrated that GTSE1 could serve as a novel and potential marker for the prognosis of breast cancer.

Our functional studies demonstrated that GTSE1 could promote the growth of breast cancer cells, which is in line with the data on HCC [18]. In addition, our data demonstrated that GTSE1 may affect the AKT pathway to facilitate breast cancer cells growth. Moreover, our data also indicated that GTSE1 could enhance breast cancer cells metastasis by regulating EMT, which is in line with the data on HCC [17]. Inhibiting AKT pathway has no effect on cell metastasis, which may be due to the compensation of other tumor signaling pathways. It has also been reported that the up-regulated expression of GTSE1 in gastric cancer cells has a contribution to cisplatin resistance [19]. Importantly, our data implicated that GTSE1 downregulation contributed to an increase in sensitivity to chemotherapy-induced apoptosis after treatments with Taxol, 5-fluorouracil and Adriamycin, while the overexpression of GTSE1 exhibited opposing effects. Since GTSE1 could promote the proliferative ability of breast cancer cells, we found that it had no function on breast cancer cells spontaneous apoptosis but affected the cell cycle distribution. GTSE1 is necessary for chromosome arrangement and progression to mitosis [48]. Silencing GTSE1 significantly increased the percentage of S phase owing to the inhibition of mitosis resulting to an S phase arrest in breast cancer cells, meanwhile, the overexpression of GTSE1 increased the percentage of cells in the G2 peak, indicating that overexpression of GTSE1 delayed the M-to-G2 phase transition of breast cancer cells, which is consistent to previous study [14]. However, since GTSE1 affects breast cancer differentiation without affecting self-renewal ability, we assumed that GTSE1 may regulate the differentiation of breast cancer cells by influencing other biological processes such as EMT and cell cycle.

It has been reported that GTSE1 is one of the target genes of p53, which may also serve as a negative regulator of p53. Previous studies have demonstrated that GTSE1 may influence the cell cycle distribution by translocating p53 from the nucleus to the cytoplasm for degradation, and can downregulate p53 protein level [13]. P21 is one of the most important downstream target genes of p53 [49]. P21 protein is known as the cell cycle inhibitory protein with the most extensive kinase inhibition activity, which binds to the Cyclin-CDK complex and inhibits its activation. By inhibiting the activation of CyclinD1-CDK4/6 and CyclinE1-CDK2, p21 can prevent the phosphorylation of Rb protein and the release of transcriptional factors E2F, causing cell-cycle arrest and affecting the distribution of cell cycle [50–52]. Our findings revealed that a novel GTSE1 function in regulating cell cycle distribution. GTSE1 has no effect on a mutant p53 homozygote but had an effect on the wild-type p53 homozygote and mutant p53 heterozygote. In cells expressing the mutant p53 heterozygote, the role of GTSE1 in regulating the p53 function was mainly dependent on the wild-type portion of the p53 mutant heterozygote. We hypothesized that GTSE1 regulates the p53 function to alter the cell cycle distribution mainly by the following three ways. First, in MCF7 cells, GTSE1 played a role in a p53-dependent manner, which interacted with the wild-type p53 protein to form a complex in the nucleus and transported the p53 to the cytoplasm for degradation resulting to p53 downregulation, which in return downregulated the p21 protein level meanwhile upregulated CyclinD1 and CyclinE1 protein levels. Second, in MDA-MB-231 cells, GTSE1 bonded with the wild-type part protein of p53 mutant heterozygotes to modulate the downstream protein levels, playing a role in a p53-partial-dependent manner. Third, in MDA-MB-468 cells, GTSE1 plays a role in a p53-independent manner, which does not bind to the mutant p53 protein and had no function on it as well as its downstream proteins. Interestingly, Bublik et al found that GTSE1 protects p21 from proteasome-dependent degradation in a p53-independent mechanism, and p21 protein levels altered in accordance with GTSE1 expression [53]. Since GTSE1 is an oncogene and p21 is a tumor suppressor gene, our research showed that the variation trend of GTSE1 was opposite to that of p21, and GTSE1 regulates p21 protein level dependent on the mutation state of p53 and such regulation of p21 levels depend on the changes of the cell cycle.

**Fig. 8:**
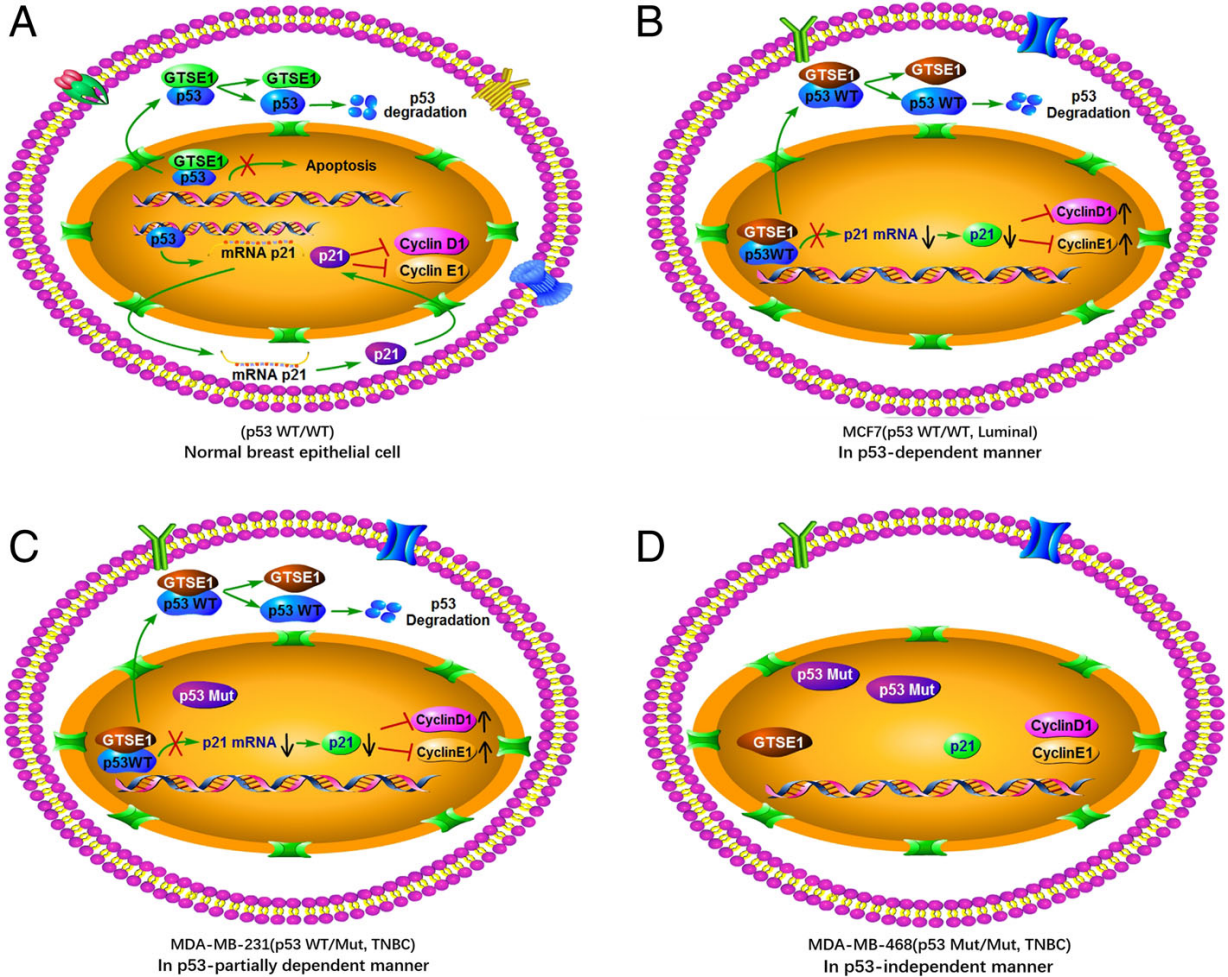
Model of the GTSE1 mechanism in breast cancer**. a** GTSE1 and p21 were the target gene of p53, and GTSE1 could inhibit the function of p53. **b** In MCF7 cells with wild type homozygote of p53, GTSE1 played a role in a p53 dependent manner. **c** In MDA-MB-231 cells with mutant heterozygote of p53, GTSE1 played a role in a p53 partial dependent manner. **d** In MDA-MB-468 cells with mutant homozygote of p53, GTSE1 played a role in a p53 independent manner

Our novel findings are summarized in Fig. 8. These findings unveiled a possible interaction between GTSE1 and p53 with different mutational status. GTSE1 regulates p53 function to promote breast cancer progression mainly by the following three ways. In MCF7 cell, tumor suppressive function of wild type p53 was restrained by oncogene GTSE1 via downregulating p53 protein level; in MDA-MB-468 cell, mutant homozygote of p53 was able to drive tumorigenesis and GTSE1 promotes cancer process in a p53-independent manner mainly by affecting stability of tubulin or EMT pathway; in MDA-MB-231 cell, GTSE1 can inhibit the function of the wild-type part protein of p53 mutant heterozygotes to promote breast cancer progression in a p53-partially dependent manner. However, we have insufficient evidence that GTSE1 cannot bind with mutant p53, in addition, GTSE1 cannot be identified as an independent prognostic indicator for breast cancer due to lack of sufficiently clinical evidence. Therefore, the role of GTSE1 should be explored further as a candidate marker for the prognosis of breast cancer, and signaling pathway research will enhance our understanding of its function in the progression of breast cancer.

## Conclusions

In summary, GTSE1 have effects on regulation of breast cancer growth and metastasis. Moreover, GTSE1 confers multi-drug resistance in breast cancer cells, and can regulate p53 function to alter cell cycle distribution dependent on p53 mutational status.

## Additional files

Additional file 1: **Table S1**: Primer sequence. **Table S2**: The sequences of small interfering RNA of GTSE1. **Table S3**: GTSE1 ‘SureSilencing shRNA’ plasmids sequences. (DOCX 27 kb)

Additional file 2: The GTSE1 protein expression level with clinical and pathological characteristics in 90 TNBC patients. (XLSX 15 kb)

Additional file 3: The GTSE1 protein expression level with clinical and pathological characteristics in 64 non-TNBC patients. (XLSX 14 kb)

Additional file 4: **Figure S1.** (A, B and C) OS, RFS and DMFS K-M curve for breast cancer patients with either GTSE1 high or GTSE1 low mRNA expression based on PROGgeneV2 database. (PDF 1460 kb)

Additional file 5: **Figure S2.** (A and B) Plate clone formation assays were conducted in the indicated cells. The data are presented in triplicates as the mean ± S.D. (PDF 4660 kb)

Additional file 6: **Figure S3.** (A) Overexpression of GTSE1 could promote cell migration in MCF7 cells compared with control cells. (B and C) Silencing or overexpression of GTSE1 markedly changed cell migration as detected by wound-healing assay. (D and E) Quantification data for C and D. *p < 0.05, *p < 0.01, ***p < 0.001, t-test. (F and G) The invasion assays were conducted in the indicated cell lines for a specific AKT inhibitor LY294002. (H) Western blot analysis of EMT maker in indicated cells after LY294002 treatment, and GAPDH as a loading control. (I) The survival data from PROGgeneV2 database shows that lung metastasis-free survival was reduced in the GTSE1 high expression group. (PDF 16315 kb)

Additional file 7: **Figure S4.** (A and B) The gating of MDA-MB-231 siNC and siGTSE1 group cells were treated with 5-fluorouracil (5μg/ml) for 48 h and test for apoptosis respectively. (C and D) The gating of MDA-MB-468 siNC and siGTSE1 group cells were treated with 5-fluorouracil (0.9μg/ml) for 48 h and test for apoptosis respectively. (E and F) The gating of MDA-MB-231-vector and overexpression of GTSE1 group cells were treated with 5-fluorouracil (5μg/ml) for 48 h, then test its apoptosis rate respectively. (G and H) The gating of MCF7-vector and overexpression of GTSE1 group cells were treated with 5-fluorouracil (20μg/ml) for 48 h, then test its apoptosis rate respectively. (PDF 3207 kb)

Additional file 8: **Figure S5.** (A) Pattern of β-Galactosidase staining in the indicated cell. (B) The data are presented as the mean ± S.D. **p < 0.01, Student’s t-test. (from triplicates). (PDF 2428 kb)

## Abbreviations

ATCC: American Type Culture Collection
DMEM: Dulbecco’s modified Eagle’s medium
EMT: Epithelial-mesenchymal transition
FBS: Fetal bovine serum
FDR: False discovery rate
GTSE1: G2 and S phase-expressed-1
H&E: Hematoxylin and eosin
IHC: Immuno-histochemical
NPI: Nottingham prognostic index
OS: Overall survival
PCR: Polymerase chain reaction
RFS: Recurrence-free survival
RNAi: RNA interference
SBR: Scarff-Bloom-Richardson
siRNA: Small interfering RNA
TCGA: The Cancer Genome Atlas
TNBC: Triple negative breast cancer
