# Supplementary material for: GTSE1 is involved in breast cancer progression in p53 mutation-dependent manner
Source: J Exp Clin Cancer Res. 2019 Apr 8;38:152. doi: 10.1186/s13046-019-1157-4 (PMC6454633; doi:10.1186/s13046-019-1157-4)
Supplement: Supplementary file 1 — Table S1: Primer sequence. Table S2: The sequences of small interfering RNA of GTSE1. Table S3: GTSE1 ‘SureSilencing shRNA’ plasmids sequences. (DOCX 27 kb) [file 13046_2019_1157_MOESM1_ESM.docx]

**Additional file 1: Table S1**

| Primer | Sequence |
| --- | --- |
| GTSE1-F | CTCTACCAGCAATCTCGCAAGG |
| GTSE1-R | GACTTGCTGATGTTTGACAGAGG |
| GAPDH-F | GTCTCCTCTGACTTCAACAGCG |
| GAPDH-R | ACCACCCTGTTGCTGTAGCCAA |

**Additional file 1: Table S2**

|  | sense（5'-3'） | antisense（5'-3'） |
| --- | --- | --- |
| GTSE1-Homo-272 | GGAUGACCCUAAGAAGGAATT | UUCCUUCUUAGGGUCAUCCTT |

**Additional file 1: Table S3**

| **Suggested Sequencing Primers:** |
| --- |
| Forward: 5'-TAATACGACTCACTATAGGG-3' |
| Reverse: 5'-CTGGAATAGCTCAGAGGC-3' |

| >HSH012794-34-LVRU6P |
| --- |
| GAATTCTCGACCTCGAGCTGTGGAATGTGTGTCAGTTAGGGTGTGGAAAGTCCCCAGGCT |
| CCCCAGCAGGCAGAAGTATGCAAAGCATGCATCTCAATTAGTCAGCAACCAGGTGTGGAA |
| AGTCCCCAGGCTCCCCAGCAGGCAGAAGTATGCAAAGCATGCATCTCAATTAGTCAGCAA |
| CCATAGTCCCGCCCCTAACTCCGCCCATCCCGCCCCTAACTCCGCCCAGTTCCGCCCATT |
| CTCCGCCCCATGGCTGACTAATTTTTTTTATTTATGCAGAGGCCGAGGCCGCCTCTGCCT |
| CTGAGCTATTCCAGAAGTAGTGAGGAGGCTTTTTTGGAGGCCTAGGCTTTTGCAAAAAGC |
| TCCCGGGAGCTTGTATATCCATTTTCGGATCTGATCGGCGCGCCATACCGGTCGCCACCA |
| TGACCGAGTACAAGCCCACGGTGCGCCTCGCCACCCGCGACGACGTCCCCCGGGCCGTAC |
| GCACCCTCGCCGCCGCGTTCGCCGACTACCCCGCCACGCGCCACACCGTCGACCCGGACC |
| GCCACATCGAGCGGGTCACCGAGCTGCAAGAACTCTTCCTCACGCGCGTCGGGCTCGACA |
| TCGGCAAGGTGTGGGTCGCGGACGACGGCGCCGCGGTGGCGGTCTGGACCACGCCGGAGA |
| GCGTCGAAGCGGGGGCGGTGTTCGCCGAGATCGGCCCGCGCATGGCCGAGTTGAGCGGTT |
| CCCGGCTGGCCGCGCAGCAACAGATGGAAGGCCTCCTGGCGCCGCACCGGCCCAAGGAGC |
| CCGCGTGGTTCCTGGCCACCGTCGGCGTCTCGCCCGACCACCAGGGCAAGGGTCTGGGCA |
| GCGCCGTCGTGCTCCCCGGAGTGGAGGCGGCCGAGCGCGCCGGGGTGCCCGCCTTCCTGG |
| AGACCTCCGCGCCCCGCAACCTCCCCTTCTACGAGCGGCTCGGCTTCACCGTCACCGCCG |
| ACGTCGAGGTGCCCGAAGGACCGCGCACCTGGTGCATGACCCGCAAGCCCGGTGCCTAGA |
| CGCGTCTGGAACAATCAACCTCTGGATTACAAAATTTGTGAAAGATTGACTGGTATTCTT |
| AACTATGTTGCTCCTTTTACGCTATGTGGATACGCTGCTTTAATGCCTTTGTATCATGCT |
| ATTGCTTCCCGTATGGCTTTCATTTTCTCCTCCTTGTATAAATCCTGGTTGCTGTCTCTT |
| TATGAGGAGTTGTGGCCCGTTGTCAGGCAACGTGGCGTGGTGTGCACTGTGTTTGCTGAC |
| GCAACCCCCACTGGTTGGGGCATTGCCACCACCTGTCAGCTCCTTTCCGGGACTTTCGCT |
| TTCCCCCTCCCTATTGCCACGGCGGAACTCATCGCCGCCTGCCTTGCCCGCTGCTGGACA |
| GGGGCTCGGCTGTTGGGCACTGACAATTCCGTGGTGTTGTCGGGGAAGCTGACGTCCTTT |
| CCATGGCTGCTCGCCTGTGTTGCCACCTGGATTCTGCGCGGGACGTCCTTCTGCTACGTC |
| CCTTCGGCCCTCAATCCAGCGGACCTTCCTTCCCGCGGCCTGCTGCCGGCTCTGCGGCCT |
| CTTCCGCGTCTTCGCCTTCGCCCTCAGACGAGTCGGATCTCCCTTTGGGCCGCCTCCCCG |
| CCTGGAATTAATTCTGCAGTCGAGACCTAGAAAAACATGGAGCAATCACAAGTAGCAATA |
| CAGCAGCTACCAATGCTGATTGTGCCTGGCTAGAAGCACAAGAGGAGGAGGAGGTGGGTT |
| TTTCCAGTCACACCTCAGGACCTTTAAGACCAATGACTTACAAGGCAGCTGTAGATCTTA |
| GCCACTTTTTAAAAGAAAAGAGGGGACTGGAAGGGCTAATTCACTCCCAACGAAGACAAG |
| ATCTGCTTTTTGCCTGTACTGGGTCTCTCTGGTTAGACCAGATCTGAGCCTGGGAGCTCT |
| CTGGCTAACTAGGGAACCCACTGCTTAAGCCTCAATAAAGCTTGCCTTGAGTGCTTCAAG |
| TAGTGTGTGCCCGTCTGTTGTGTGACTCTGGTAACTAGAGATCCCTCAGACCCTTTTAGT |
| CAGTGTGGAAAATCTCTAGCAGTAGTAGTTCATGTCATCTTATTATTCAGTATTTATAAC |
| TTGCAAAGAAATGAATATCAGAGAGTGAGAGGCTAGCGTTTTACCGTCGACCTCTAGCTA |
| GAGCTTGGCGTAATCATGGTCATAGCTGTTTCCTGTGTGAAATTGTTATCCGCTCACAAT |
| TCCACACAACATACGAGCCGGAAGCATAAAGTGTAAAGCCTGGGGTGCCTAATGAGTGAG |
| CTAACTCACATTAATTGCGTTGCGCTCACTGCCCGCTTTCCAGTCGGGAAACCTGTCGTG |
| CCAGCTGCATTAATGAATCGGCCAACGCGCGGGGAGAGGCGGTTTGCGTATTGGGCGCTC |
| TTCCGCTTCCTCGCTCACTGACTCGCTGCGCTCGGTCGTTCGGCTGCGGCGAGCGGTATC |
| AGCTCACTCAAAGGCGGTAATACGGTTATCCACAGAATCAGGGGATAACGCAGGAAAGAA |
| CATGTGAGCAAAAGGCCAGCAAAAGGCCAGGAACCGTAAAAAGGCCGCGTTGCTGGCGTT |
| TTTCCATAGGCTCCGCCCCCCTGACGAGCATCACAAAAATCGACGCTCAAGTCAGAGGTG |
| GCGAAACCCGACAGGACTATAAAGATACCAGGCGTTTCCCCCTGGAAGCTCCCTCGTGCG |
| CTCTCCTGTTCCGACCCTGCCGCTTACCGGATACCTGTCCGCCTTTCTCCCTTCGGGAAG |
| CGTGGCGCTTTCTCATAGCTCACGCTGTAGGTATCTCAGTTCGGTGTAGGTCGTTCGCTC |
| CAAGCTGGGCTGTGTGCACGAACCCCCCGTTCAGCCCGACCGCTGCGCCTTATCCGGTAA |
| CTATCGTCTTGAGTCCAACCCGGTAAGACACGACTTATCGCCACTGGCAGCAGCCACTGG |
| TAACAGGATTAGCAGAGCGAGGTATGTAGGCGGTGCTACAGAGTTCTTGAAGTGGTGGCC |
| TAACTACGGCTACACTAGAAGAACAGTATTTGGTATCTGCGCTCTGCTGAAGCCAGTTAC |
| CTTCGGAAAAAGAGTTGGTAGCTCTTGATCCGGCAAACAAACCACCGCTGGTAGCGGTTT |
| TTTTGTTTGCAAGCAGCAGATTACGCGCAGAAAAAAAGGATCTCAAGAAGATCCTTTGAT |
| CTTTTCTACGGGGTCTGACGCTCAGTGGAACGAAAACTCACGTTAAGGGATTTTGGTCAT |
| GAGATTATCAAAAAGGATCTTCACCTAGATCCTTTTAAATTAAAAATGAAGTTTTAAATC |
| AATCTAAAGTATATATGAGTAAACTTGGTCTGACAGTTACCAATGCTTAATCAGTGAGGC |
| ACCTATCTCAGCGATCTGTCTATTTCGTTCATCCATAGTTGCCTGACTCCCCGTCGTGTA |
| GATAACTACGATACGGGAGGGCTTACCATCTGGCCCCAGTGCTGCAATGATACCGCGAGA |
| CCCACGCTCACCGGCTCCAGATTTATCAGCAATAAACCAGCCAGCCGGAAGGGCCGAGCG |
| CAGAAGTGGTCCTGCAACTTTATCCGCCTCCATCCAGTCTATTAATTGTTGCCGGGAAGC |
| TAGAGTAAGTAGTTCGCCAGTTAATAGTTTGCGCAACGTTGTTGCCATTGCTACAGGCAT |
| CGTGGTGTCACGCTCGTCGTTTGGTATGGCTTCATTCAGCTCCGGTTCCCAACGATCAAG |
| GCGAGTTACATGATCCCCCATGTTGTGCAAAAAAGCGGTTAGCTCCTTCGGTCCTCCGAT |
| CGTTGTCAGAAGTAAGTTGGCCGCAGTGTTATCACTCATGGTTATGGCAGCACTGCATAA |
| TTCTCTTACTGTCATGCCATCCGTAAGATGCTTTTCTGTGACTGGTGAGTACTCAACCAA |
| GTCATTCTGAGAATAGTGTATGCGGCGACCGAGTTGCTCTTGCCCGGCGTCAATACGGGA |
| TAATACCGCGCCACATAGCAGAACTTTAAAAGTGCTCATCATTGGAAAACGTTCTTCGGG |
| GCGAAAACTCTCAAGGATCTTACCGCTGTTGAGATCCAGTTCGATGTAACCCACTCGTGC |
| ACCCAACTGATCTTCAGCATCTTTTACTTTCACCAGCGTTTCTGGGTGAGCAAAAACAGG |
| AAGGCAAAATGCCGCAAAAAAGGGAATAAGGGCGACACGGAAATGTTGAATACTCATACT |
| CTTCCTTTTTCAATATTATTGAAGCATTTATCAGGGTTATTGTCTCATGAGCGGATACAT |
| ATTTGAATGTATTTAGAAAAATAAACAAATAGGGGTTCCGCGCACATTTCCCCGAAAAGT |
| GCCACCTGACGTCGACGGATCGGGAGATCAACTTGTTTATTGCAGCTTATAATGGTTACA |
| AATAAAGCAATAGCATCACAAATTTCACAAATAAAGCATTTTTTTCACTGCATTCTAGTT |
| GTGGTTTGTCCAAACTCATCAATGTATCTTATCATGTCTGGATCAACTGGATAACTCAAG |
| CTAACCAAAATCATCCCAAACTTCCCACCCCATACCCTATTACCACTGCCAATTACCCTG |
| TGGGCGCAATTAACCCTCACTAAAGGGAACAAAAGCTGGAGCTGCAAGCTTAATGTAGTC |
| TTATGCAATACTCTTGTAGTCTTGCAACATGGTAACGATGAGTTAGCAACATGCCTTACA |
| AGGAGAGAAAAAGCACCGTGCATGCCGATTGGTGGAAGTAAGGTGGTACGATCGTGCCTT |
| ATTAGGAAGGCAACAGACGGGTCTGACATGGATTGGACGAACCACTGAATTGCCGCATTG |
| CAGAGATATTGTATTTAAGTGCCTAGCTCGATACATAAACGGGTCTCTCTGGTTAGACCA |
| GATCTGAGCCTGGGAGCTCTCTGGCTAACTAGGGAACCCACTGCTTAAGCCTCAATAAAG |
| CTTGCCTTGAGTGCTTCAAGTAGTGTGTGCCCGTCTGTTGTGTGACTCTGGTAACTAGAG |
| ATCCCTCAGACCCTTTTAGTCAGTGTGGAAAATCTCTAGCAGTGGCGCCCGAACAGGGAC |
| TTGAAAGCGAAAGGGAAACCAGAGGAGCTCTCTCGACGCAGGACTCGGCTTGCTGAAGCG |
| CGCACGGCAAGAGGCGAGGGGCGGCGACTGGTGAGTACGCCAAAAATTTTGACTAGCGGA |
| GGCTAGAAGGAGAGAGATGGGTGCGAGAGCGTCAGTATTAAGCGGGGGAGAATTAGATCG |
| CGATGGGAAAAAATTCGGTTAAGGCCAGGGGGAAAGAAAAAATATAAATTAAAACATATA |
| GTATGGGCAAGCAGGGAGCTAGAACGATTCGCAGTTAATCCTGGCCTGTTAGAAACATCA |
| GAAGGCTGTAGACAAATACTGGGACAGCTACAACCATCCCTTCAGACAGGATCAGAAGAA |
| CTTAGATCATTATATAATACAGTAGCAACCCTCTATTGTGTGCATCAAAGGATAGAGATA |
| AAAGACACCAAGGAAGCTTTAGACAAGATAGAGGAAGAGCAAAACAAAAGTAAGACCACC |
| GCACAGCAAGCGGCCGGCCGCTGATCTTCAGACCTGGAGGAGGAGATATGAGGGACAATT |
| AATTGGAGAAGTGAATTATATAAATATAAAGTAGTAAAAATTGAACCATTAGGAGTAGCA |
| CCCACCAAGGCAAAGAGAAGAGTGGTGCAGAGAGAAAAAAGAGCAGTGGGAATAGGAGCT |
| TTGTTCCTTGGGTTCTTGGGAGCAGCAGGAAGCACTATGGGCGCAGCGTCAATGACGCTG |
| ACGGTACAGGCCAGACAATTATTGTCTGGTATAGTGCAGCAGCAGAACAATTTGCTGAGG |
| GCTATTGAGGCGCAACAGCATCTGTTGCAACTCACAGTCTGGGGCATCAAGCAGCTCCAG |
| GCAAGAATCCTGGCTGTGGAAAGATACCTAAAGGATCAACAGCTCCTGGGGATTTGGGGT |
| TGCTCTGGAAAACTCATTTGCACCACTGCTGTGCCTTGGAATGCTAGTTGGAGTAATAAA |
| TCTCTGGAACAGATTTGGAATCACACGACCTGGATGGAGTGGGACAGAGAAATTAACAAT |
| TACACAAGCTTAATACACTCCTTAATTGAAGAATCGCAAAACCAGCAAGAAAAGAATGAA |
| CAAGAATTATTGGAATTAGATAAATGGGCAAGTTTGTGGAATTGGTTTAACATAACAAAT |
| TGGCTGTGGTATATAAAATTATTCATAATGATAGTAGGAGGCTTGGTAGGTTTAAGAATA |
| GTTTTTGCTGTACTTTCTATAGTGAATAGAGTTAGGCAGGGATATTCACCATTATCGTTT |
| CAGACCCACCTCCCAACCCCGAGGGGACCCGACAGGCCCGAAGGAATAGAAGAAGAAGGT |
| GGAGAGAGAGACAGAGACAGATCCATTCGATTAGTGAACGGATCTCGACGGTATCGCCTT |
| TAAAAGAAAAGGGGGGATTGGGGGGTACAGTGCAGGGGAAAGAATAGTAGACATAATAGC |
| AACAGACATACAAACTAAAGAATTACAAAAACAAATTACAAAAATTCAAAATTTTCGGGT |
| TTATTACAGGGACAGCAGAGATCCAGTTTATCTAATACGACTCACTATAGGGAGAGAGAG |
| AGAATTACCCTCACTAAAGGGAGGAGAAGCATGAATTGTCCCCAGTGGAAAGACGCGCAG |
| GCAAAACGCACCACGTGACGGAGCGTGACCGCGCGCCGAGCGCGCGCCAAGGTCGGGCAG |
| GAAGAGGGCCTATTTCCCATGATTCCTTCATATTTGCATATACGATACAAGGCTGTTAGA |
| GAGATAATTAGAATTAATTTGACTGTAAACACAAAGATATTAGTACAAAATACGTGACGT |
| AGAAAGTAATAATTTCTTGGGTAGTTTGCAGTTTTAAAATTATGTTTTAAAATGGACTAT |
| CATATGCTTACCGTAACTTGAAAGTATTTCGATTTCTTGGGTTTATATATCTTGTGGAAA |
| GGACGAGgatccgggctcttcttgtagatatcaatcaagagttgatatctacaagaagag |
| ccttttttg |

**Additional file 1: Table S4**

| **Suggested Sequencing Primers:** |
| --- |
| Forward: 5'-GCGGTAGGCGTGTACGGT-3' |
| Reverse: 5'-ATTGTGGATGAATACTGCC-3' |

| **ORF Sequence Information for EX-Y4953-Lv121** |
| --- |
| >EX-Y4953-Lv121 ORF sequence |
| ATGGAAGGAGGCGGCGGCCGCGATGAGCCTTCAGCCTGCCGGGCAGGGGACGTGAACATGGATGACCCTAAGAAGGAAGA |
| CATTCTTCTTTTGGCCGATGAAAAATTTGACTTCGATCTTTCATTGTCTTCTTCGAGTGCAAATGAAGATGATGAAGTCT |
| TCTTCGGACCCTTTGGACATAAAGAAAGATGTATTGCTGCCAGCTTGGAATTAAATAATCCGGTTCCCGAACAGCCTCCG |
| TTGCCCACATCTGAGAGTCCCTTTGCCTGGAGCCCTCTGGCCGGGGAGAAGTTCGTGGAGGTGTACAAAGAAGCTCACTT |
| ACTGGCTTTACACATTGAGAGCAGCAGCCGGAACCAGGCAGCCCAAGCTGCCAAGCCTGAAGACCCTCGGAGCCAGGGCG |
| TGGAAAGATTCATACAGGAGTCAAAATTAAAAATAAACCTCTTTGAGAAAGAAAAGGAAATGAAGAAAAGCCCCACGTCT |
| CTTAAAAGGGAGACATACTACCTGTCAGACAGCCCCTTGCTGGGGCCCCCTGTGGGTGAGCCTCGGCTCTTGGCCTCCTC |
| CCCGGCCCTGCCCAGCTCTGGTGCCCAGGCCCGCCTCACCCGGGCGCCGGGGCCTCCGCACTCTGCTCATGCTTTGCCCA |
| GGGAATCATGCACTGCTCATGCTGCAAGTCAGGCAGCGACTCAGAGGAAGCCCGGGACCAAATTGCTGCTGCCTCGAGCG |
| GCCTCTGTTAGAGGAAGAAGCATCCCTGGGGCTGCGGAGAAGCCCAAGAAAGAGATTCCAGCTAGTCCTTCCAGGACAAA |
| AATCCCAGCTGAGAAGGAATCCCACCGGGATGTTCTCCCTGACAAACCTGCCCCGGGTGCTGTCAATGTGCCGGCCGCCG |
| GAAGCCACTTGGGCCAGGGCAAGCGGGCGATCCCTGTTCCAAACAAGTTGGGGCTGAAGAAGACCCTGTTAAAAGCACCC |
| GGCTCTACCAGCAATCTCGCAAGGAAGTCCTCCTCGGGGCCTGTTTGGAGCGGGGCATCCAGTGCGTGCACATCCCCAGC |
| AGTGGGCAAAGCTAAATCAAGTGAATTTGCAAGTATTCCTGCAAATAGCTCCCGGCCTCTGTCAAACATCAGCAAGTCAG |
| GCAGAATGGGACCCGCCATGCTGCGGCCAGCTCTGCCTGCAGGCCCTGTGGGGGCATCCTCCTGGCAGGCCAAGCGGGTC |
| GATGTTTCTGAGCTGGCAGCGGAGCAGCTCACGGCACCCCCCTCAGCATCCCCCACCCAACCCCAGACTCCGGAAGGTGG |
| CGGCCAGTGGCTGAACTCCAGTTGCGCTTGGTCAGAATCTTCTCAATTGAATAAGACTAGAAGTATCAGACGGCGAGATT |
| CCTGTCTAAATTCCAAGACAAAGGTTATGCCTACTCCTACAAATCAATTTAAAATTCCTAAGTTTTCTATTGGTGACTCC |
| CCGGACAGCTCAACACCAAAGCTTTCGCGGGCACAGCGGCCGCAGTCGTGCACGTCAGTTGGCAGGGTCACTGTCCACAG |
| CACCCCGGTTAGACGCTCATCTGGGCCAGCACCACAAAGCCTGCTGAGCGCACGGCGTGTGTCAGCCTTGCCCACACCCG |
| CCAGCCGGCGCTGCTCTGGCCTTCCACCGATGACCCCCAAAACGATGCCCAGGGCCGTGGGCTCTCCCCTGTGTGTGCCA |
| GCTCGGAGACGTTCCTCTGAGCCCCGCAAGAACTCTGCAATGAGAACTGAACCAACAAGGGAGAGCAACAGAAAGACAGA |
| TTCCAGGCTGGTGGATGTGTCCCCTGACAGGGGTTCTCCTCCTTCCCGTGTGCCTCAGGCACTTAACTTTTCTCCAGAGG |
| AAAGCGATTCTACTTTCTCCAAAAGTACTGCCACAGAAGTAGCTCGGGAGGAAGCCAAGCCGGGTGGAGATGCAGCCCCT |
| AGTGAGGCTCTTCTTGTAGATATCAAACTGGAACCACTCGCGGTCACTCCAGATGCTGCAAGCCAGCCCCTCATTGACCT |
| TCCTCTCATCGACTTCTGCGATACCCCAGAAGCACACGTGGCTGTAGGATCTGAAAGCAGGCCTCTGATCGACCTCATGA |
| CAAACACTCCAGACATGAATAAAAATGTGGCCAAACCTTCACCGGTGGTGGGACAGCTCATAGACCTGAGCTCCCCTCTG |
| ATCCAGCTGAGCCCTGAGGCTGACAAGGAGAACGTGGATTCCCCACTCCTCAAGTTCTAC |
