# Supplementary figures and images for: GTSE1 is involved in breast cancer progression in p53 mutation-dependent manner
Source: J Exp Clin Cancer Res. 2019 Apr 8;38:152. doi: 10.1186/s13046-019-1157-4 (PMC6454633; doi:10.1186/s13046-019-1157-4)

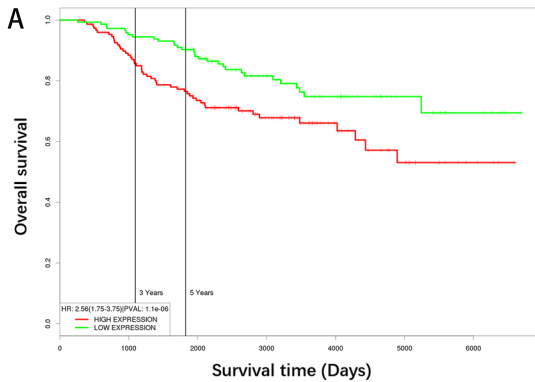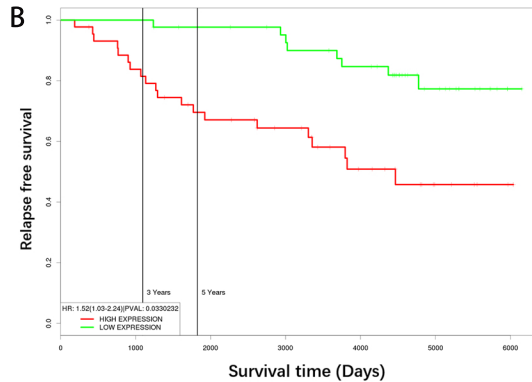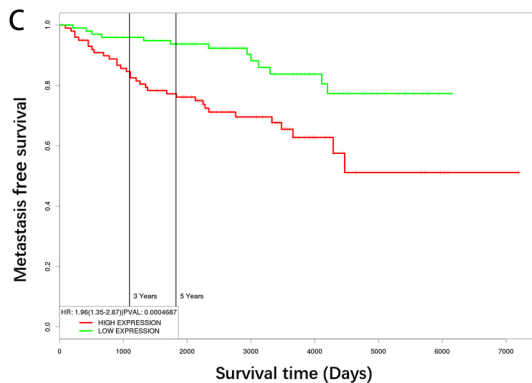

Supplement: Supplementary file 4 — Figure S1. (A, B and C) OS, RFS and DMFS K-M curve for breast cancer patients with either GTSE1 high or GTSE1 low mRNA expression based on PROGgeneV2 database. (PDF 1460 kb) [file 13046_2019_1157_MOESM4_ESM.pdf]

A

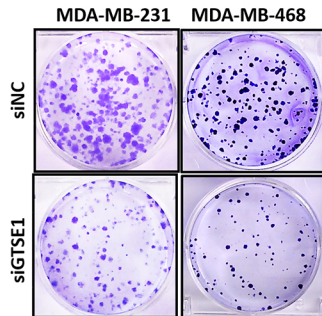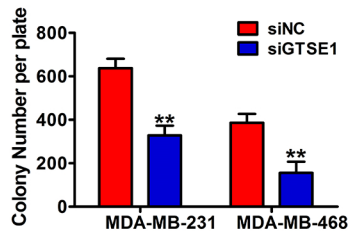

B

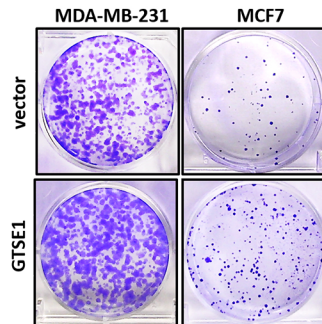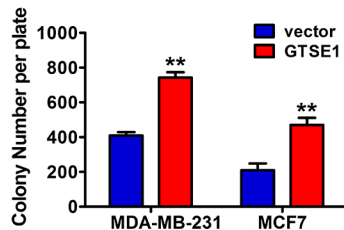

Supplement: Supplementary file 5 — Figure S2. (A and B) Plate clone formation assays were conducted in the indicated cells. The data are presented in triplicates as the mean ± S.D. (PDF 4660 kb) [file 13046_2019_1157_MOESM5_ESM.pdf]

A

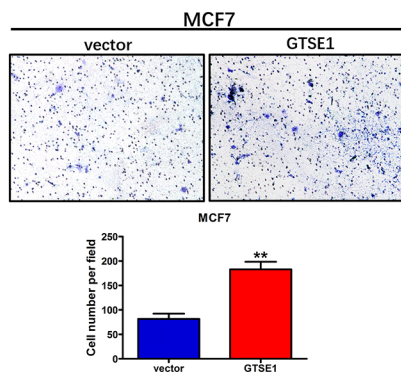

B

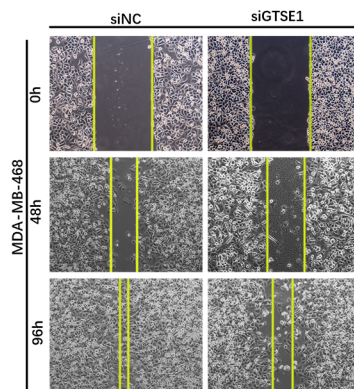

C

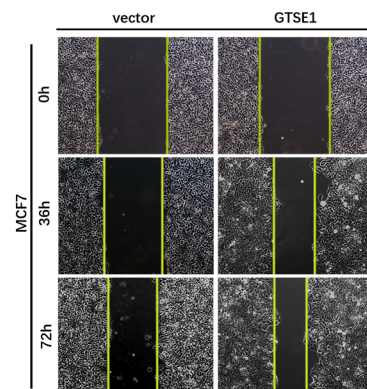

D

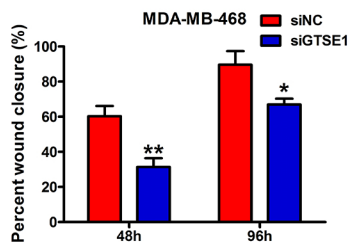

E

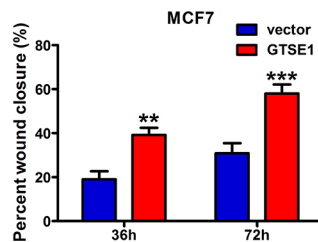

F

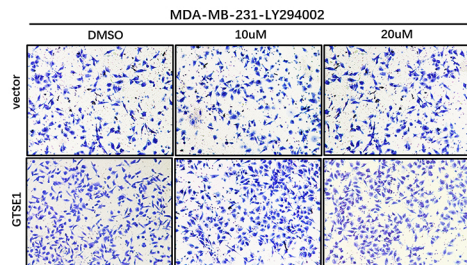

G

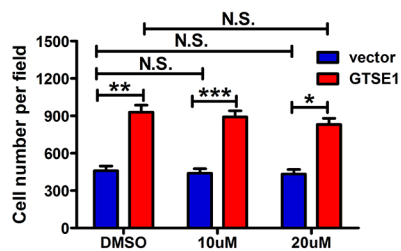

H

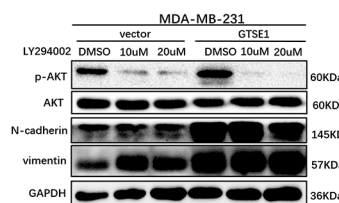

I

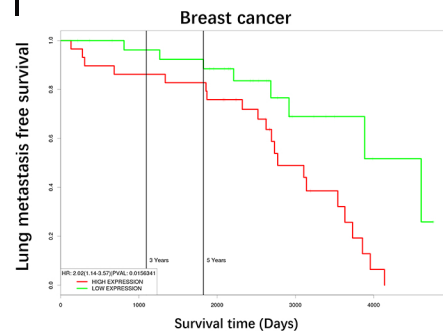

Supplement: Supplementary file 6 — Figure S3. (A) Overexpression of GTSE1 could promote cell migration in MCF7 cells compared with control cells. (B and C) Silencing or overexpression of GTSE1 markedly changed cell migration as detected by wound-healing assay. (D and E) Quantification data for C and D. *p < 0.05, *p < 0.01, ***p < 0.001, t-test. (F and G) The invasion assays were conducted in the indicated cell lines for a specific AKT inhibitor LY294002. (H) Western blot analysis of EMT maker in indicated cells after LY294002 treatment, and GAPDH as a loading control. (I) The survival data from PROGgeneV2 database shows that lung metastasis-free survival was reduced in the GTSE1 high expression group. (PDF 16315 kb) [file 13046_2019_1157_MOESM6_ESM.pdf]

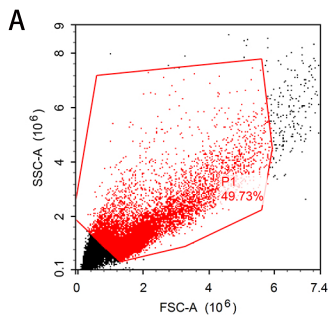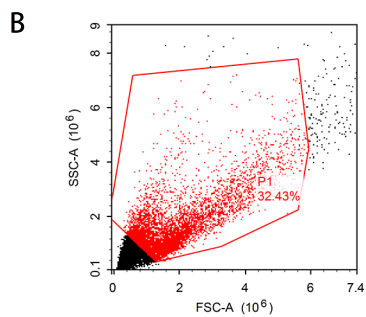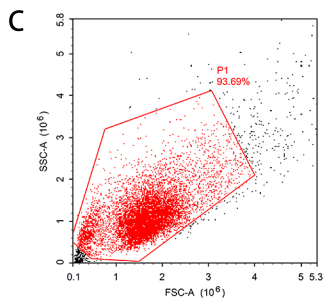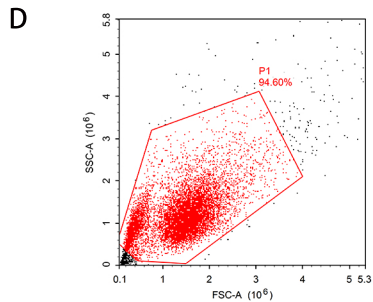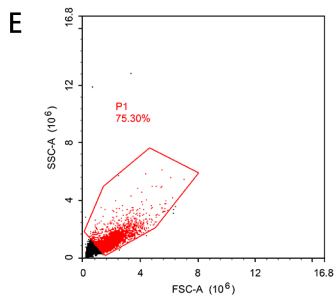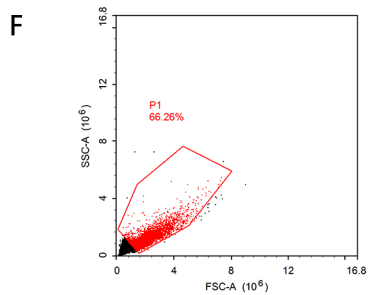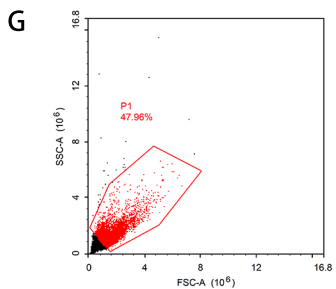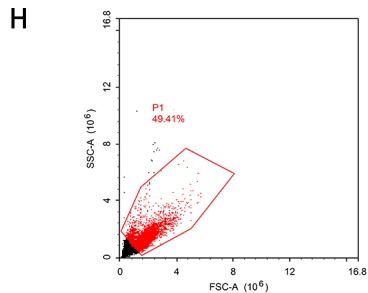

Supplement: Supplementary file 7 — Figure S4. (A and B) The gating of MDA-MB-231 siNC and siGTSE1 group cells were treated with 5-fluorouracil (5μg/ml) for 48 h and test for apoptosis respectively. (C and D) The gating of MDA-MB-468 siNC and siGTSE1 group cells were treated with 5-fluorouracil (0.9μg/ml) for 48 h and test for apoptosis respectively. (E and F) The gating of MDA-MB-231-vector and overexpression of GTSE1 group cells were treated with 5-fluorouracil (5μg/ml) for 48 h, then test its apoptosis rate respectively. (G and H) The gating of MCF7-vector and overexpression of GTSE1 group cells were treated with 5-fluorouracil (20μg/ml) for 48 h, then test its apoptosis rate respectively. (PDF 3207 kb) [file 13046_2019_1157_MOESM7_ESM.pdf]

A

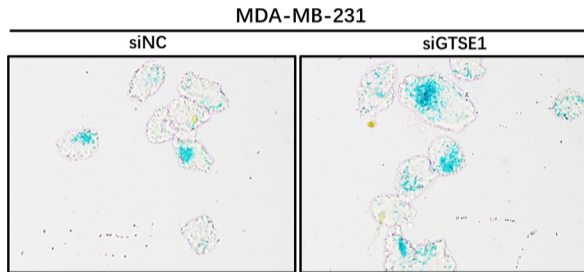

B

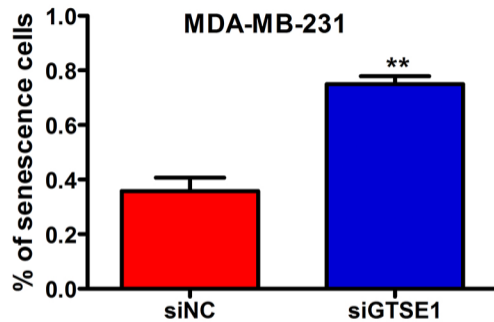

Supplement: Supplementary file 8 — Figure S5. (A) Pattern of β-Galactosidase staining in the indicated cell. (B) The data are presented as the mean ± S.D. **p < 0.01, Student’s t-test. (from triplicates). (PDF 2428 kb) [file 13046_2019_1157_MOESM8_ESM.pdf]
